# Local translational program at the muscle-tendon junction endows domain identity in muscle syncytia

**DOI:** 10.1126/sciadv.aee6166

**Published:** 2026-11-04

**Authors:** Nour El Khazen, Juliana de Carvalho Neves, Vedran Franke, Coalesco Smith, Sakulrat Mankhong, Edgar Jauliac, Laura Yedigaryan, Elise Lefebvre, Altuna Akalin, Pascal Maire, Minchul Kim

**Affiliations:** ^1^ Laboratory of Syncytial Cell Biology, IGBMC, Institute of Genetics and of Molecular and Cellular Biology, 67400 Illkirch, France.; ^2^ Université de Strasbourg, Strasbourg, France.; ^3^ CNRS, UMR 7104, 67400 Illkirch, France.; ^4^ INSERM, UMR-S 1258, 67400 Illkirch, France.; ^5^ Berlin Institute for Medical Systems Biology, Max Delbrück Center for Molecular Medicine, Helmholtz Society, Berlin, Germany.; ^6^ Université Paris Cité, CNRS, Inserm, Institut Cochin, F-75014 Paris, France.; ^7^ Laboratory of Pathophysiology of Neuromuscular Diseases, IGBMC, Institute of Genetics and of Molecular and Cellular Biology, 67400 Illkirch, France.

## Abstract

How cells establish specialized subdomains is a fundamental question in cell and tissue biology. Skeletal muscle fibers, among the largest cells in the body, are multinucleated and form distinct regions such as the neuromuscular and myotendinous junctions (MTJs), the latter forming a critical interface between muscle and tendon that transmits contractile force. While transcriptional heterogeneity among myonuclei has been described, whether local translation contributes to domain identity remains unknown, largely due to the lack of tools for domain-specific manipulation. Here, we introduce MTJ-AAV, a viral system that enables selective genetic targeting of MTJ myonuclei. This approach allowed MTJ-specific ribosome tagging and revealed extensive translational regulation underlying MTJ biology and its remodeling during exercise. Untranslated regions of these transcripts were sufficient to control regionalized translation. Notably, the Krüpper-like factor–family transcription factors emerged as translationally up-regulated targets at the MTJ, where they drive local gene expression. Our findings establish local translation as a key layer of subcellular specialization and provide a versatile toolkit for dissecting spatial molecular regulation within muscle syncytia.

## INTRODUCTION

To precisely coordinate diverse biochemical activities and biological functions, cells must establish specialized subcellular domains. How these domains form and how they are maintained and remodeled remain a fundamental question in cell biology. Skeletal muscle cells, or myofibers, provide a unique model to study this process because of their exceptionally large cytoplasm, which contains hundreds to thousands of nuclei. Different myofiber regions interact with distinct external cues, giving rise to specialized functional domains. For example, myofiber central regions interact with motor neurons, forming the neuromuscular junction (*1*, *2*), while their ends attach to tendons, creating the myotendinous junction (MTJ) (*3*, *4*). The MTJ plays a critical role in dissipating the mechanical forces generated during contraction and transmitting them to tendons. Consequently, the MTJ is highly susceptible to injury, and its dysfunction leads to muscle rupture (*5*). Understanding how these muscle domains are regulated not only advances our knowledge of myology and disease, but also provides broader insights into how cells spatially and functionally organize their intracellular space.

Previous studies using single-nucleus RNA sequencing (snRNA-seq) have shown that muscle nuclei at specialized domains adopt distinct gene expression programs, conceptually resembling cellular differentiation in multicellular tissues (*6*–*9*). These studies highlight transcriptional control as an important mechanism for domain formation. However, the contribution of posttranscriptional regulation remains largely unexplored. Translational regulation, the control of protein synthesis from existing mRNA, has emerged as a central mechanism across diverse biological contexts, including gametogenesis, neurodevelopment, cancer, and inflammatory signaling (*10*–*14*). In muscle, translational profiling has thus far been limited to whole myofibers (*15*–*17*), leaving little insight into how localized protein synthesis supports muscle domain biology. Although the MTJ undergoes structural remodeling during exercise and aging (*18*, *19*), snRNA-seq studies have not detected major transcriptomic changes in MTJ myonuclei (*7*, *20*). This discrepancy suggests that posttranscriptional regulation, particularly translation, may play a key role in these adaptations.

Conventional genetic approaches in the muscle field lack spatial resolution and target all myonuclei indiscriminately. The distinct lack of genetic tools that can manipulate specific domains is the major roadblock preventing the field from studying regionalized molecular events in myofibers. To overcome this limitation, we reasoned that promoters of MTJ-specific genes identified from snRNA-seq datasets could be leveraged to drive gene expression specifically within this domain.

Here, we develop a versatile adeno-associated virus (AAV) system that enables expression of genetic tools specifically at the MTJ. Using this platform, we profile the local translational landscapes of the MTJ and uncover extensive translational regulation that becomes markedly amplified during endurance exercise. We further demonstrate that untranslated regions (UTRs) of some MTJ-enriched transcripts are sufficient to confer localized translation and that Krüpper-like factor (KLF) transcription factors are translationally up-regulated to drive local gene expression. Together, these findings reveal a previously unrecognized layer of spatial regulation underlying this specialized muscle domain and provide a powerful toolkit for dissecting subcellular regulation in a syncytium.

## RESULTS

### Development of MTJ-AAV

To selectively target the MTJ, we focused on *Tigd4*, a highly specific marker of MTJ nuclei (*6*). We synthesized a fragment of the *Tigd4* promoter, spanning from the transcription start site to −3.3 kb, and cloned it into an AAV plasmid to drive the expression of super-fold GFP (sfGFP) fused with four SV40-derived nuclear localization signals (NLSs). The 3.3-kb length was chosen to accommodate AAV genome size constraints. The resulting pAAV-Tigd4-NLS-sfGFP construct was packaged into the MyoAAV4a serotype, which has previously been shown to selectively transduce myofibers (*21*) and intramuscularly injected into 10-week-old wild-type (WT) mice (Fig. 1A). As a control, we performed parallel experiments using the conventional *CK8* promoter, which is active in all myonuclei.

**Fig. 1.**
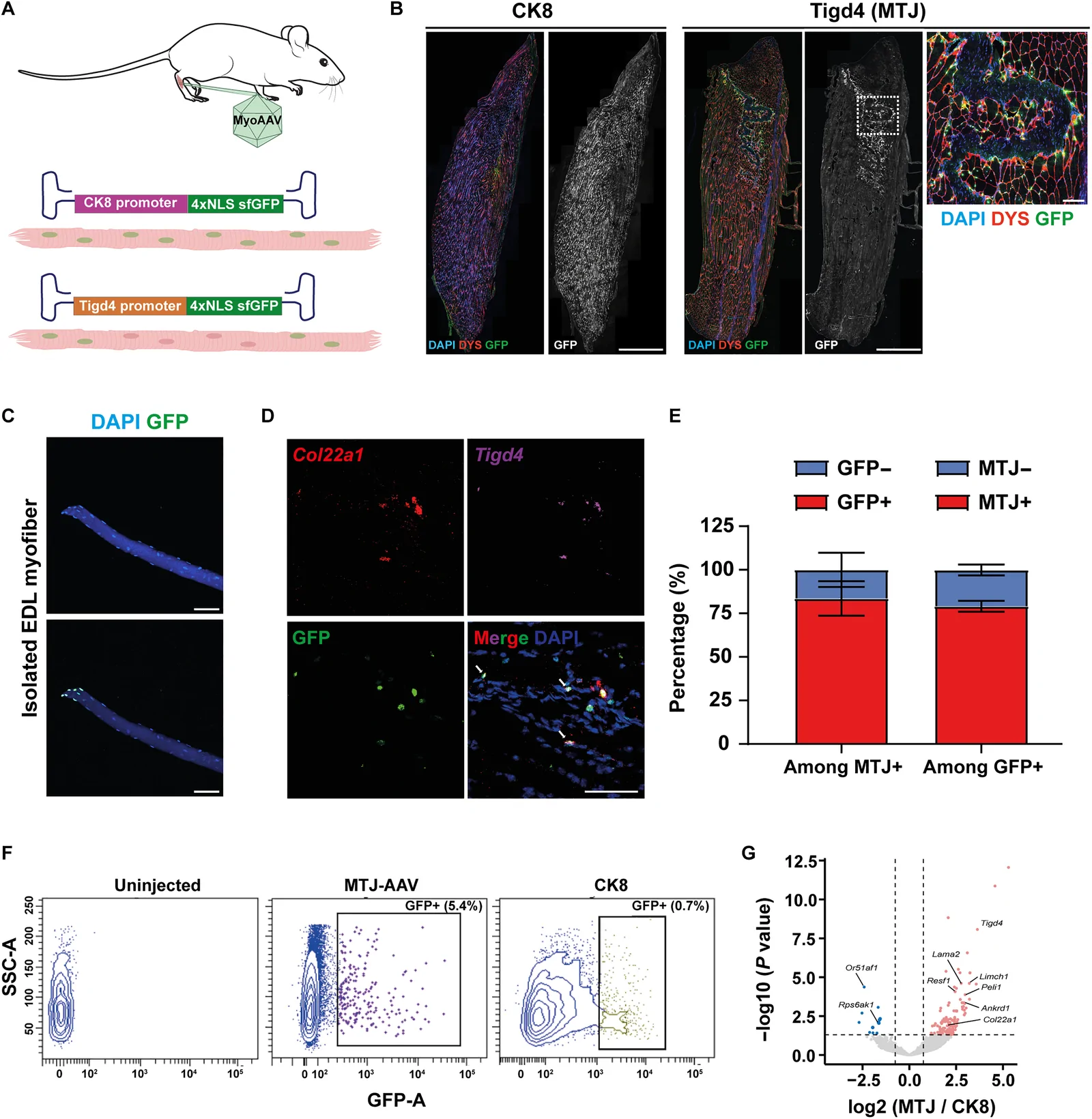
Development of MTJ-AAV. (**A**) Schematic of AAV constructs driven by *CK8* or *Tigd4* promoters and the expected expression pattern of the 4×NLS-sfGFP reporter. (**B**) Tile-scan images of tibialis anterior (TA) muscles injected with the AAVs shown in (A). The right panel shows a magnified view of the boxed region. Scale bars, 1000 μm for the tile scan images and 100 μm for the inset. (**C**) Single myofibers were isolated from the extensor digitorum longus (EDL) muscle of MTJ-AAV–injected mice and stained with DAPI. Scale bar, 50 μm. (**D**) Combined GFP immunostaining and RNAscope detection of *Col22a1* and *Tigd4* transcripts. Arrows indicate nuclei coexpressing all three markers. Scale bar, 100 μm. (**E**) Quantification of labeling efficiency and specificity from experiments in (D) (*n* = 5). Error bars indicate SEM. (**F**) Representative FACS plots of CK8- and Tigd4-NLS-sfGFP–injected TA muscles. GFP^+^ nuclei were isolated and subjected to bulk RNA-seq (*n* = 3 for MTJ and *n* = 4 for CK8). (**G**) Volcano plot showing genes enriched or depleted in GFP^+^ nuclei labeled by the *Tigd4* promoter relative to the *CK8* promoter. Selected MTJ marker genes are indicated.

As expected, *CK8* promoter–driven AAV broadly labeled the entire muscle tissue with NLS-sfGFP when tibialis anterior (TA) muscles were examined 1-month postinjection. In contrast, the *Tigd4* promoter restricted reporter expression to the MTJ, identifiable by characteristic 4′,6-diamidino-2-phenylindole (DAPI)–stained structures penetrating into the muscle together with Dystrophin staining outlining the myofiber boundaries (Fig. 1B). Visualization of GFP in isolated extensor digitorum longus (EDL) myofibers similarly showed clear enrichment at the fiber tips (Fig. 1C and fig. S1). Quantification of labeling efficiency using GFP immunostaining combined with RNAscope for the MTJ markers *Tigd4* and *Col22a1* revealed that approximately 80% of MTJ nuclei (defined by coexpression of *Tigd4* and *Col22a1*) were GFP positive (Fig. 1, D and E). Conversely, only ∼20% of GFP-positive nuclei were not MTJ nuclei, indicating high labeling specificity. This transduction efficiency is comparable to that reported in previous studies using intramuscular AAV delivery (*22*).

All GFP-positive nuclei expressed the pan-myonuclear markers *Ttn* and *Neb*, confirming that they were myonuclei (fig. S2, A and C). To further assess cellular specificity, we examined markers of neighboring non-myonuclear cell populations present at the MTJ. Previous studies identified a fibroblast-lineage population associated with the muscle tips and proposed to fuse with myofiber ends (*23*, *24*). Consistent with this idea, our previous snRNA-seq analysis identified a related population, termed MTJ-B nuclei, which expresses both muscle and fibroblast-associated genes (*6*). In that study, Ebf1 emerged as a selective marker of MTJ-B nuclei. In muscles injected with pAAV-Tigd4-NLS-sfGFP, however, GFP-positive nuclei never colocalized with Ebf1 (fig. S2, B and C). Likewise, GFP-positive nuclei did not colocalize with Pdgfra (a marker of fibro-adipogenic progenitors) (fig. S2, B and C). Furthermore, throughout these staining processes, we did not observe any GFP-positive nuclei in tenocytes located between the opposing Dystrophin-positive myofiber membranes at the MTJ. Together, these findings demonstrate that the *Tigd4* promoter specifically labels MTJ myonuclei.

To confirm the molecular identity of the labeled nuclei via an orthogonal method, we isolated GFP-positive nuclei by fluorescence-activated cell sorting (FACS; Fig. 1F) and performed bulk RNA-seq (Fig. 1G). On average, we isolated around 1000 MTJ myonuclei from a single TA muscle. The analysis showed many representative MTJ markers, including *Tigd4*, *Col22a1*, *Resf1*, *Lama2*, and *Ankrd1*, to be enriched in GFP-positive nuclei from the *Tigd4* promoter–driven AAV compared to those labeled by the *CK8* promoter (Fig. 1F). References to these MTJ marker genes can be found in the main text or supplementary datasets of the cited studies (*6*–*8*). From here on, we refer to our AAV system that uses *Tigd4* promoter as “MTJ-AAV.”

We next tested whether we could express other genetic tools at the MTJ as it will broaden the utility of MTJ-AAV. As such, we asked whether MTJ-restricted recombination could be achieved by delivering Cre recombinase. MTJ-AAV carrying Cre-NLS was intramuscularly injected into *Rosa26-LSL-H2B-GFP* reporter mice (Fig. 2A), resulting in GFP labeling around the MTJ (Fig. 2B). Quantification using MTJ markers showed a similar labeling efficiency of ∼75% (Fig. 2C). However, off-target labeling was higher in this model, with ∼40% of GFP-positive nuclei not corresponding to MTJ nuclei (Fig. 2C). Similar to our previous approach, all labeled nuclei were positive for the pan-myonuclear markers *Ttn* and *Neb*, and did not colocalize with Ebf1 or Pdgfra, confirming their myonuclear identity and excluding labeling of adjacent connective tissue cell populations (fig. S3).

**Fig. 2.**
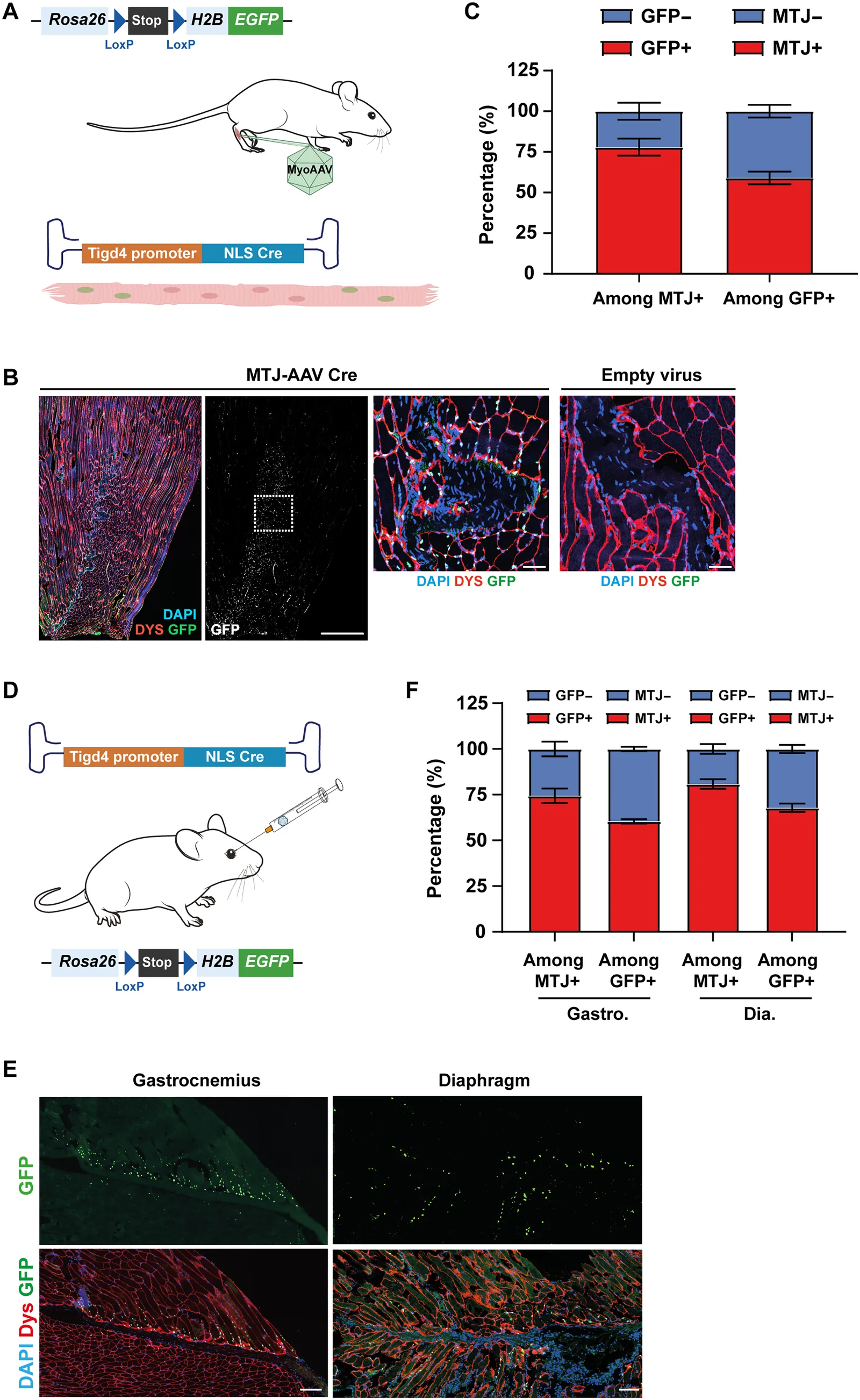
MTJ-AAV enables Cre-mediated recombination specifically at the MTJ. (**A**) Schematic of the AAV construct in which the *Tigd4* promoter drives expression of Cre-NLS. When injected into *Rosa26-LSL-H2B-GFP* reporter mice, Cre activity induces H2B-GFP labeling of MTJ nuclei. (**B**) Tile-scan images of TA muscles injected with MTJ-AAV-Cre-NLS. Reporter mice injected with an empty MyoAAV4a vector (containing only the *Tigd4* promoter without Cre) showed no H2B-GFP expression. Scale bars, 1000 μm for the tile scan image and 50 μm for the insets. (**C**) Quantification of labeling efficiency and specificity from experiments in (B) (*n* = 4). (**D**) Schematic of systemic delivery of MTJ-AAV-Cre-NLS into *Rosa26-LSL-H2B-GFP* reporter mice via intra-orbital injection. (**E**) Representative images showing MTJ-specific myonuclear labeling in the gastrocnemius and diaphragm muscles. Similar results were obtained in three independent animals. Scale bars, 100 μm. (**F**) Quantification of labeling efficiency and specificity from experiments in (E) (*n* = 3). All error bars indicate SEM.

To investigate why there was higher off-target labeling with Cre compared with NLS-sfGFP, we first tested whether Cre protein diffuses to neighboring nuclei. Cre/GFP coimmunohistochemistry revealed that only ∼56% of GFP-positive nuclei exhibited a detectable Cre signal (fig. S4, A and B). Given the ∼40% off-target labeling, this result suggests that Cre protein remains largely restricted to MTJ nuclei with minimal diffusion, although we cannot rule out the possibility that undetectable amounts of Cre protein contribute to leaky recombination. Next, we tested whether H2B-GFP protein is more diffusive than the NLS-sfGFP protein used earlier by performing a side-by-side comparison. Expressing H2B-GFP via MTJ-AAV resulted in a similar ∼80% labeling efficiency but a higher off-target labeling rate of ∼40% compared to ∼20% with NLS-sfGFP (fig. S4C). These findings indicate that diffusion properties of expressed factors can vary and require careful assessment before downstream experiments.

Last, we tested whether MTJ-AAV could be delivered systemically (Fig. 2D). Intra-orbital injection of MTJ-AAV carrying Cre-NLS into the H2B-GFP reporter successfully labeled MTJ nuclei across multiple muscles, including the gastrocnemius and diaphragm (Fig. 2E). Quantification of labeling efficiency and specificity showed values comparable to those from intramuscular injection above (Fig. 2F).

### Genetic labeling of MTJ ribosomes and translational profiling

Although protein synthesis is central to cellular identity and function, regionalized translation at the MTJ has not been explored, largely due to the absence of suitable tools. We hypothesized that combining MTJ-AAV with genetic ribosome tagging would enable selective profiling of ribosome-associated transcripts.

We therefore intramuscularly injected MTJ-AAV-Cre-NLS or CK8-Cre-NLS into *LSL-HA-Rpl22* (RiboTag) mice (*25*), in which Cre-mediated recombination introduces an HA tag into the endogenous Rpl22 ribosomal subunit (Fig. 3A). HA immunohistochemistry confirmed the tight expression of HA-Rpl22 at the MTJ (Fig. 3B). We also performed the same experiment in Ribo-Trap mice (*Rosa26-LSL-GFP-L10a*) (*26*) and observed similar results. However, GFP-L10a displayed broader expression than HA-Rpl22 (fig. S5), likely due to its overexpression from the *Rosa26* locus. For this reason, we focused on the RiboTag model for subsequent analysis.

**Fig. 3.**
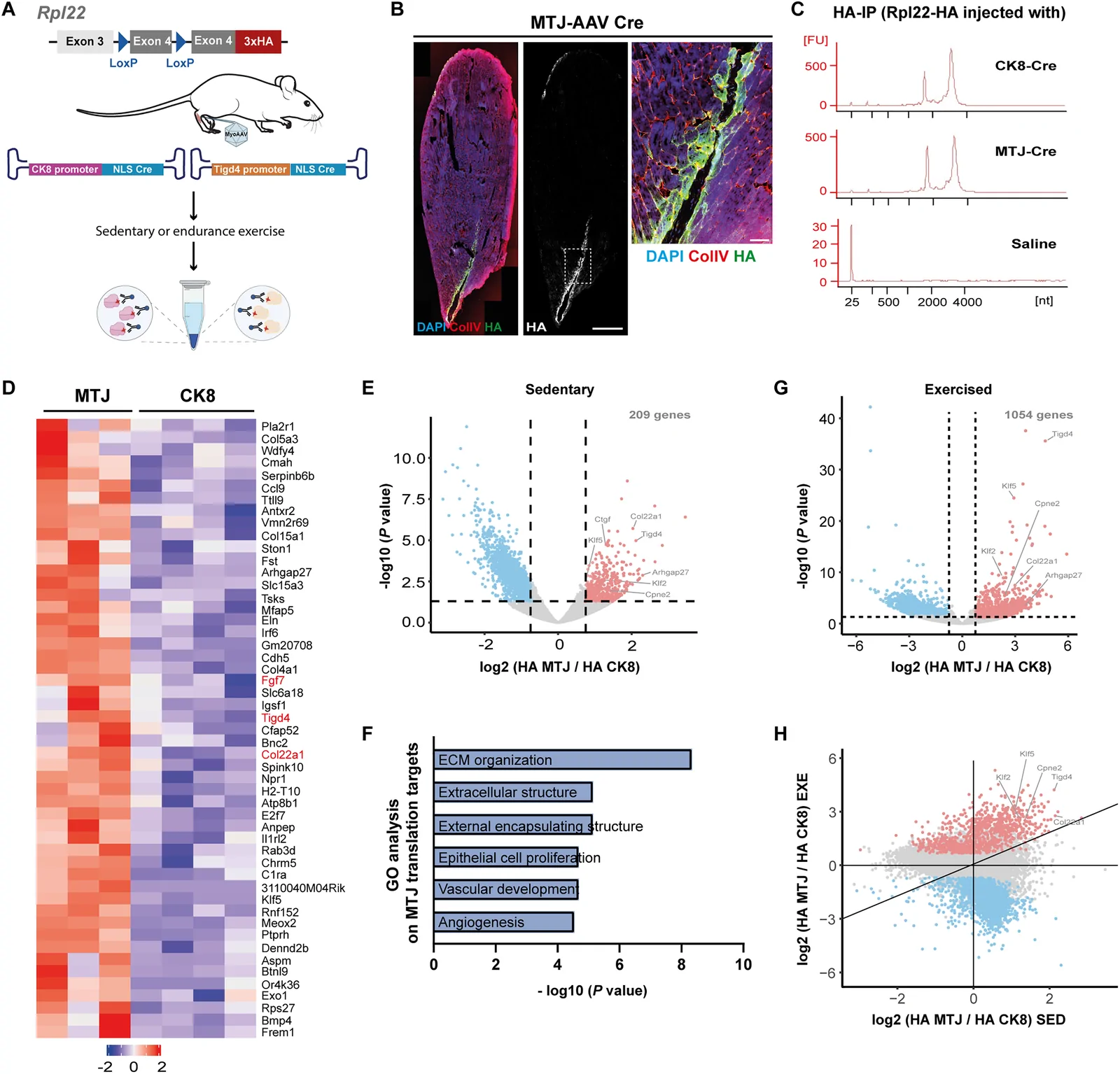
MTJ-AAV reveals a specialized translatome of MTJ. (**A**) Experimental workflow for MTJ-targeted ribosome labeling and translatome profiling in sedentary and exercised muscles. For CK8-Cre samples, two TA muscles from different mice were pooled to generate each biological replicate, whereas four TA muscles from different mice were pooled for each MTJ-Cre replicate. The number of biological replicates was *n* = 4 for sedentary CK8-Cre, *n* = 3 for sedentary MTJ-Cre, *n* = 3 for exercised CK8-Cre, and *n* = 3 for exercised MTJ-Cre. (**B**) HA immunohistochemistry confirming ribosome labeling along the MTJ. The right panel shows a magnified view of the boxed region in the middle image. Scale bars, 1000 μm for the tile scan image and 100 μm for the inset. (**C**) Bioanalyzer profile of RNA recovered after HA immunoprecipitation. FU, fluorescence unit. (**D**) Heatmap showing representative MTJ ribosome–enriched transcripts under sedentary conditions. Known transcriptional MTJ markers are indicated in red. (**E**) Volcano plot comparing CK8- and MTJ ribosome–associated transcripts in sedentary muscles. (**F**) Gene Ontology analysis of translationally enriched MTJ targets. (**G**) Volcano plot comparing CK8- and MTJ ribosome–associated transcripts following endurance exercise. (**H**) Exercise amplifies the degree of translational enrichment for MTJ-specific targets relative to sedentary conditions.

To isolate ribosome-associated transcripts, TA tissue lysates were subjected to anti-HA immunoprecipitation. Bioanalyzer profiles confirmed robust RNA recovery from Cre-delivered RiboTag muscles, with no detectable RNA in saline-injected controls (Fig. 3C). RNA-seq was then performed to map the MTJ translational landscape (*n* = 4 for whole muscle and *n* = 3 for MTJ). Notably, MTJ markers *Tigd4* and *Col22a1* were among the most enriched ribosome-associated transcripts, validating the specificity of our dataset (Fig. 3, D and E).

Notably, ∼80% of MTJ ribosome–enriched transcripts (170 of 209; fold change >2, *P* < 0.05) have not been identified as transcriptionally enriched in published snRNA-seq datasets when compared manually across reported MTJ marker lists (*6*–*8*). To evaluate the contribution of translational regulation more quantitatively, we generated a scatter plot comparing MTJ enrichment in our ribosome immunoprecipitation and sequencing (RiboTag-seq) dataset with MTJ enrichment in published snRNA-seq data (*7*) (fig. S6A). This analysis revealed substantial discordance between the translatome and transcriptome. In particular, 91.3% of MTJ ribosome–enriched genes showed greater enrichment at the ribosome level than at the transcript level (red genes located to the left of the *y* = *x* line in fig. S6A).

The heatmap in fig. S6B further illustrates the relative contributions of translational and transcriptional regulation. Among the 304 genes enriched in MTJ-associated ribosomes in this categorization, only 30 were also transcriptionally enriched by snRNA-seq, and previously identified MTJ marker genes from two published snRNA-seq studies (*6*, *7*) were heavily concentrated within this groups. The complete gene list is provided in table S2.

Consistent with MTJ function, Gene Ontology (GO) analysis of genes preferentially enriched in the MTJ translatome showed enrichment for those related to extracellular matrix biology, cytoskeletal organization, and plasma membrane structure (Fig. 3F). Intriguingly, angiogenesis-related genes were also enriched, though the importance of this remains unclear. Manual inspection identified additional targets of diverse function, including the E3 ligases *Klhl42*, *Rnf152*, and *Rnf43* and transcription factors such as *Klf2* and *Klf5*. GO analysis of genes enriched primarily at the transcript level only (snRNA-seq) in MTJ revealed a distinct set of categories such as regulation of intracellular transport and membrane potential (fig. S6C). This suggests that the canonical role of the MTJ as a mechanical buffer might be more prominently reflected in the translatome than in the transcriptome. Conversely, translationally repressed genes were mainly linked to mitochondria and sarcoplasmic reticulum biology (fig. S6, D and E), suggesting distinct organelle composition or density at the MTJ.

We also note that translational specialization may not be unique to the MTJ. A comparable fraction (92.6%) of CK8 ribosome-enriched genes showed the opposite pattern, namely, greater enrichment at the translatome than at the transcriptome level (orange genes located to the right of the *y* = *x* line in fig. S6A). These observations raise the possibility that distinct regions of the syncytial muscle cell maintain specialized translational programs. Nonetheless, the extent to which this occurs should be interpreted with caution because direct comparison between bulk RiboTag-seq and snRNA-seq datasets is inherently limited. Consequently, the exact position of the *y* = *x* line in fig. S6A should not be regarded as absolute.

### Rewiring of translational program upon sustained exercise

Endurance exercise remodels the MTJ by increasing the depth and branching of its interdigitated structures (*18*). To examine whether this event involves translational control, we subjected whole-muscle and MTJ ribosome–tagged mice to an endurance training regimen, followed by RiboTag-seq (Fig. 3A).

We first identified a set of genes that were translationally induced throughout the muscle in response to exercise, mainly being inflammatory mediators (*Nfkbia*, *Nfkb2*, *Stat4*, and *Socs3*) (fig. S7A). Notably, many of these were also induced in the MTJ translatome upon exercise, indicating a genuine muscle-wide response (fig. S7B). Pathway analysis of genes up-regulated in both CK8 and MTJ datasets revealed strong enrichment for cytokine-related signaling pathways (fig. S7C).

We next compared MTJ ribosome–enriched transcripts to CK8-labeled ribosomes in exercised muscles (Fig. 3G). The number of MTJ-enriched genes increased substantially under exercise (1054 versus 209 in sedentary conditions), with significant overlap between the two sets (fig. S7D). Even shared targets exhibited markedly stronger enrichment after training (Fig. 3H). For instance, *Cpne2* increased from a 1.3-fold enrichment (log_2_ scale) at rest to 2.4-fold after training, while *Klf2* rose from 1.32- to 2.29-fold. In addition, many new genes were identified to be specifically enriched in MTJ-associated ribosome after exercise such as *Gys2*, *Vipr2*, and *Robo4* (see table S3 for the full list).

GO analysis of MTJ ribosome–enriched genes in exercised muscles identified biological processes largely similar to those observed under sedentary conditions, including muscle contraction, extracellular matrix organization, and circulation-related pathways (fig. S7E). In contrast, analysis of MTJ ribosome–depleted genes revealed a distinct pattern. Whereas terms related to intracellular organelles predominated under sedentary conditions (fig. S6E), genes associated with neurotransmission became the dominant category following exercise (fig. S7F). The biological implication of this shift remains unclear. Together, these findings demonstrate that the MTJ translatome is significantly regulated by exercise, amplifying a preexisting local translational program to meet the heightened mechanical demands of sustained activity.

### Role of UTRs in conferring translational specificity

Next, we asked how certain transcripts are selected for local translation. UTRs often contain regulatory elements that control where and how efficiently mRNAs are translated (*27*, *28*). To test whether such elements mediate local translation at the MTJ, we generated translation reporters based on MTJ-enriched genes. We selected *Arhgap27* and *Cpne2* as models because they were not previously identified as transcriptional markers of MTJ myonuclei but showed consistent enrichment in our RiboTag-seq profiling as indicated in the volcano plots in Fig. 3 (E and G). Moreover, their UTRs are sufficiently long (∼1 kb) to harbor potential regulatory motifs yet compact enough to remain compatible with AAV packaging constraints. *Arhgap27* encodes a poorly characterized Rho GTPase–activating protein (*29*), whereas *Cpne2* encodes a similarly underexplored calcium-dependent phospholipid-binding protein that links membranes to the cytoskeleton (*30*). Both genes therefore represent plausible effectors of MTJ function, where precise coordination between cytoskeletal and membrane dynamics is essential.

We first confirmed that *Cpne2* and *Arhgap27* transcripts are uniformly distributed throughout myofibers by RNAscope (fig. S8A). To validate that Cpne2 and Arhgap27 proteins are enriched at the MTJ, we surgically fractionated MTJ and non-MTJ areas using soleus muscle. We chose this muscle type because of its flat-shaped MTJ anatomy, making the fractionation more suitable. Western blotting for Col22a1 confirmed the validity of this strategy, where Cpne2 and Arhgap27 were also found to be more abundant at the MTJ fraction (fig. S8B). We additionally confirmed the enrichment of Cpne2 at myofiber tips in isolated EDL myofiber (fig. S8C).

We constructed AAVs in which the pan-myonuclear CK8 promoter drives an NLS-sfGFP reporter either alone or fused to the 5′ and 3′UTRs of *Arhgap27* or *Cpne2* (Fig. 4A). In the absence of UTRs, the reporter protein was uniformly expressed throughout the myofiber, whereas inclusion of the UTRs resulted in markedly enriched expression at the fiber tips (Fig. 4B). RNAscope analysis confirmed that *GFP* transcripts remained uniformly distributed even in the presence of the UTRs (Fig. 4C), indicating that localization occurs at the translational level rather than through transcriptional control or mRNA transport. Therefore, the UTRs of *Cpne2* and *Arhgap27* are sufficient to confer MTJ-specific translation.

**Fig. 4.**
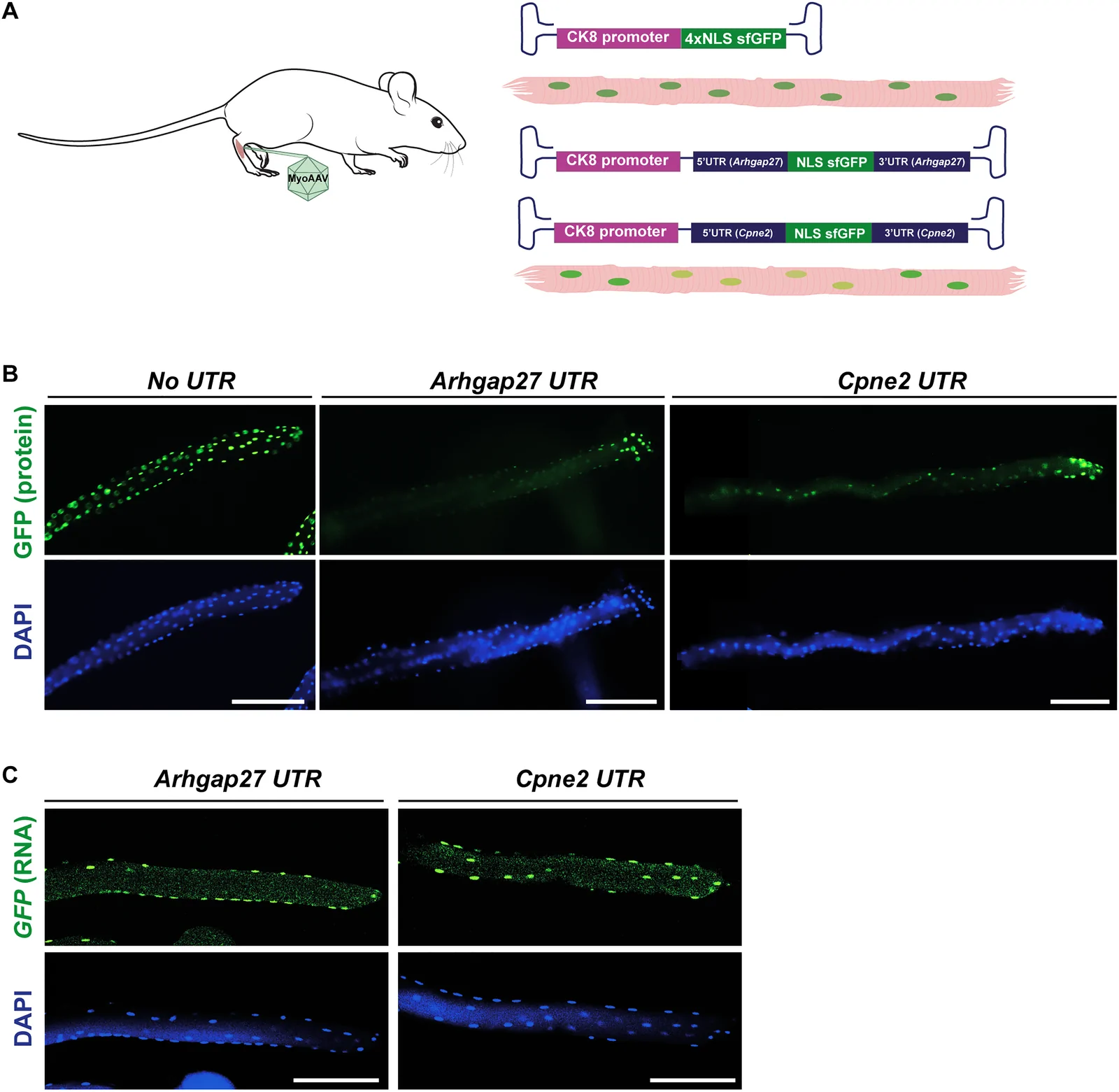
UTRs of translational target transcripts enable localized protein synthesis at the MTJ. (**A**) Schematic representation of MTJ translational reporter constructs. (**B**) Single extensor digitorum longus (EDL) myofibers were isolated 1 month after intramuscular injection of the AAVs shown in (A) and analyzed by epifluorescence microscopy. (**C**) EDL myofibers from (B) were fixed and subjected to RNAscope detection of *GFP* mRNA to assess transcript distribution. Scale bars, 100 μm.

### Translational control of KLF contributes to MTJ-specific gene expression

While previous snRNA-seq studies identified hundreds of MTJ-enriched transcripts, they did not uncover any transcription factors that are strongly and uniquely enriched at the MTJ. This contrasts with the neuromuscular junction, where key transcription factors such as Etv4 and Etv5 are specifically expressed (*6*–*9*). However, motif analysis of a published single-nucleus ATAC-seq (snATAC-seq) dataset predicted a strong enrichment of KLF-binding motifs in MTJ-specific open chromatin regions (*8*). We verified this with an independently acquired snATAC-seq dataset (*31*): 8601 MTJ-specific chromatin-accessible regions were identified that were mainly located in promoters (∼50% of all peaks) (Fig. 5A). Motif analysis of the accessible regions predicted KLF factors as the top candidates (Fig. 5B). KLFs are zinc-finger transcription factors with broad roles in development, cell identity, and extracellular matrix regulation (*32*, *33*). Although KLF family members have previously been implicated in connective tissue lineages, including tenocytes and fibroblasts during limb development (*34*, *35*), their role in MTJ myonuclei has not been explored. These analyses raise the question of how KLF factors might selectively function at the MTJ.

**Fig. 5.**
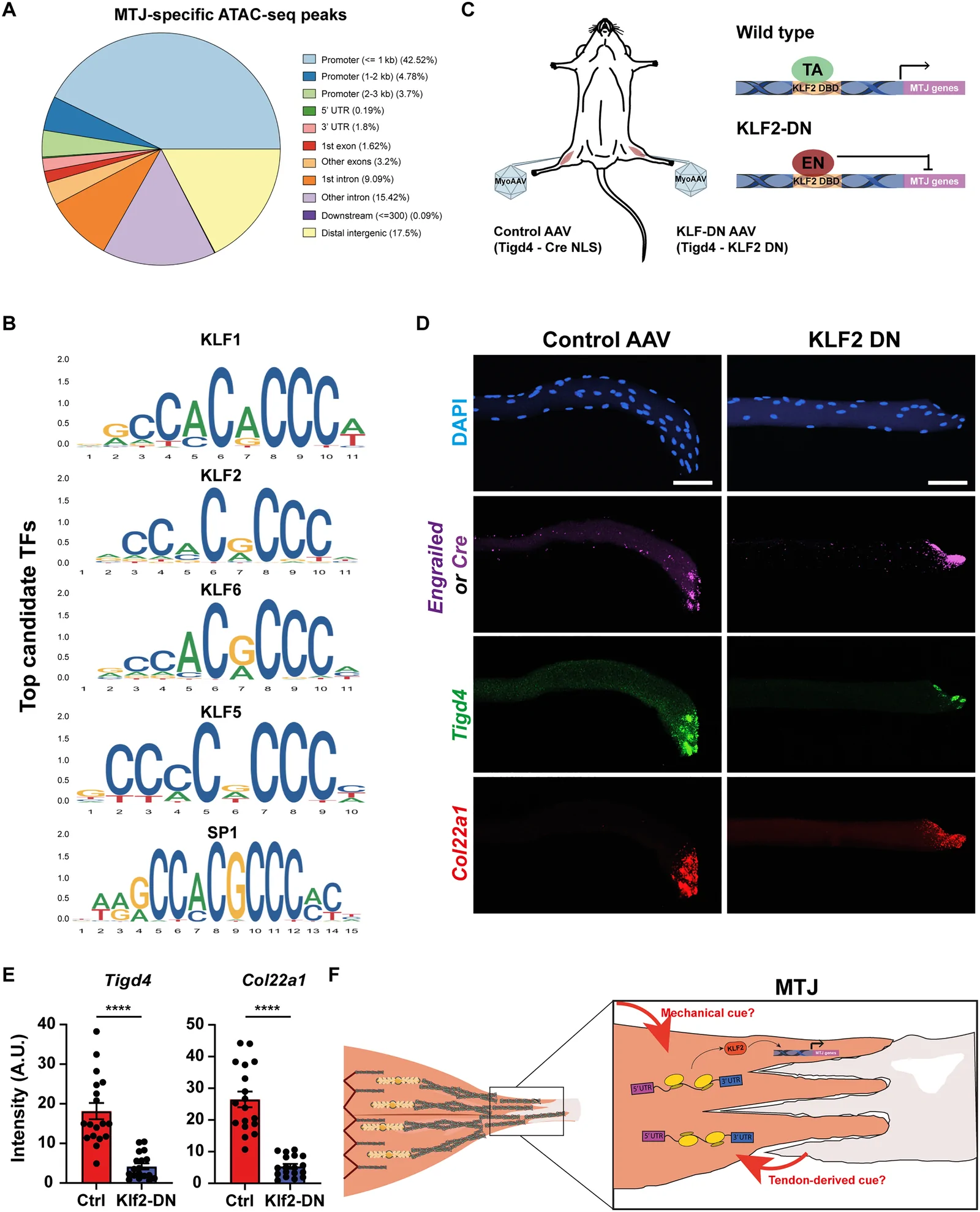
KLF transcription factors contribute to the MTJ transcriptome. (**A**) Classification of MTJ-specific ATAC-seq peaks according to their genomic locations and sequence elements. (**B**) De novo motif analysis of MTJ-specific ATAC-seq peaks identifying KLF family transcription factors as top candidates. (**C**) Schematic of KLF inhibition strategy using a dominant-negative (DN) construct expressed at the MTJ. (**D**) Expression of KLF2-DN at the MTJ suppresses the MTJ marker genes *Tigd4* and *Col22a1*. EDL myofibers were isolated and subjected to RNAscope for *Tigd4*, *Col22a1*, and *Engrailed* (to detect KLF2-DN) or *Cre* (to detect the control construct, Cre-NLS). All fiber isolation and RNAscope were done simultaneously. Scale bars, 50 μm. (**E**) Quantification of *Tigd4* and *Col22a1* RNAscope signal intensity (*n* = 18 MTJ regions collected from three control mice and three KLF2-DN–injected mice). Unpaired student’s *t* test, ****P < 0.0001. (**F**) Model summarizing MTJ-specific translational regulation and its impact on local transcriptional control through KLF factors. Local signals, such as mechanical or tendon-derived cues, activate this translational program to reinforce domain specialization.

In this light, we were intrigued to find KLF factors, *Klf2* and *Klf5*, to be enriched in our RiboTag-seq datasets of the MTJ (Fig. 3, E and G). We confirmed the enrichment of Klf2 protein at the MTJ using surgically enriched MTJ from soleus muscle (fig. S8B), as well as by immunohistochemistry on tissue sections (fig. S9A).

To probe KLF function while circumventing redundancy issue among KLF family members, we used a dominant-negative (DN) approach. We fused the DNA-binding domain of Klf2 to the transcriptional repressor domain of *Drosophila* Engrailed (Fig. 5C), a strategy previously validated in other biological contexts (*36*). For proper comparison, we intramuscularly injected a control virus (*Tigd4* promoter expressing Cre-NLS) on one hindlimb, and KLF2-DN virus on the contralateral muscle of WT mice (Fig. 5C). For clear visualization of MTJ marker transcripts, we isolated single EDL myofibers and performed RNAscope of *Cre* or *Engrailed* (to identify transduced myofibers) combined with *Tigd4* and *Col22a1*, two putative MTJ markers containing KLF motifs (fig. S9B). Targeted expression of KLF2-DN at the MTJ was clearly observed by *Engrailed* RNAscope, which effectively suppressed the expression of *Tigd4* and *Col22a1* (Fig. 5, D and E). A reduction in Col22a1 expression was also observed at the protein level by immunohistochemistry, displaying reduced signals in KLF2-DN transduced muscles (fig. S9, C and D).

To determine whether this effect extended beyond *Tigd4* and *Col22a1*, we performed snRNA-seq on WT muscles injected with MTJ-AAV expressing either Cre-NLS (control) or KLF2-DN in contralateral legs (fig. S10A). Approximately 30% of MTJ nuclei contained detectable transgene reads. The reason for this relatively low capture rate is currently unclear, but may reflect rapid export of the transgene mRNAs from the nucleus, thereby reducing their detection in snRNA-seq. Notably, we have observed a similar phenomenon in snRNA-seq experiments using myonuclear reporter mice, in which only ∼50% of reporter-positive nuclei are identified by sequence capture. Similar phenomena have also been observed by others [for example, figure 1B in (*20*)]. We therefore extracted nuclei containing detectable *Cre-NLS* or *KLF2-DN* transcripts and calculated an MTJ gene signature score based on previously published MTJ marker genes (*6*, *7*). This analysis demonstrated that KLF2-DN consistently reduced the MTJ transcriptional signature in all four animals examined (fig. S10B). Therefore, KLF transcription factors are translationally controlled at the MTJ, where they drive a downstream transcriptional program required for MTJ specialization (Fig. 5F).

## DISCUSSION

Our study establishes a framework for investigating regulatory mechanisms that allow the formation of specialized domains in multinucleated muscle fibers. By developing a viral system with MTJ specificity, we achieved domain-restricted genetic manipulations and ribosome tagging. These approaches uncovered extensive translational regulation at the MTJ. A broad range of protein are controlled by this mechanism, encompassing extracellular matrix and cytoskeletal regulators as well as transcription factors. Previous studies largely attributed the domain formation to a transcriptional specialization of local myonuclei. The results extend our understanding of how muscle domains are formed and maintained, and expand the growing appreciation that translational control represents a central regulatory layer in cell and tissue biology.

Mechanistically, our data indicate that UTRs of MTJ-enriched genes play a key role in conferring domain-restricted translation. Moreover, we show that the MTJ translational program is intensified by endurance exercise, indicating that this mechanism is dynamically regulated according to physiological demand. The precise cis-regulatory motifs and RNA-binding proteins involved remain to be identified. Future studies should determine how specific transcripts are selected for local translation (for example, through UTR motifs, RNA secondary structures, ribosome heterogeneity, or other mechanisms) and how these processes integrate with tissue-level signals at the MTJ such as tendon-derived or mechanical cues (Fig. 5F).

Of note, comparison of our RiboTag-seq dataset with a recent spatial proteomic study of the MTJ (*37*) revealed only seven overlapping factors, primarily abundant ones such as extracellular matrix, cytoskeletal, and adhesion proteins (Aoc3, Ckap4, Col4a2, Col5a1, Col5a2, Eln, and Tns2). This limited overlap is likely due to several reasons. First, proteomics preferentially detects abundant and relatively stable proteins, whereas our approach captures ribosome-associated transcripts. Second, the proteomic study analyzed serial cryosections spanning the muscle-tendon interface and therefore measured proteins derived from multiple sources including myofibers, fibroblasts, tenocytes, and extracellular matrix. Last, steady-state protein abundance reflects not only translation, but also protein stability and turnover. By contrast, MTJ-AAV–based RiboTag profiling selectively interrogates the translational landscape of the bona fide MTJ domain of myofibers, enabling the identification of low-abundance regulatory factors such as transcription factors.

We also found that KLF transcription factors are translationally up-regulated at the MTJ and contribute to local gene expression. This explains why KLF motifs are highly enriched in accessible chromatin regions of MTJ nuclei, although *Klf* transcript levels at the MTJ are similar to those observed in non-MTJ myonuclei. However, KLF factors are not strictly MTJ exclusive; they are also detectable in non-MTJ nuclei, albeit at lower levels. This suggests that KLF expression alone is insufficient to convey MTJ identity and must act in concert with additional, yet unidentified regulators to establish the full MTJ transcriptome. Our results broaden the concept that KLF factors function as master regulators of connective tissue and its associated attachment structures. During limb development, Klf2 and Klf4 promote a hybrid chondrocyte-tenocyte gene expression program that is essential for the formation of tendon-to-bone attachment sites (*34*). Genome-wide profiling of Klf2/4 binding in developing chick limb connective tissues further demonstrated their occupancy at genes involved in extracellular matrix organization and cell adhesion (*35*). Our findings extend this paradigm by suggesting that KLF factors also operate in the specialized myonuclei located on the muscle side of the muscle-tendon interface.

A key innovation of our work is the flexibility of the MTJ-AAV strategy. Beyond transcriptional and translational profiling, it can be adapted to deliver diverse genetic tools to investigate the MTJ. For example, coupling MTJ-AAV with inducible recombinases such as CreERT2 should enable long-term lineage tracing and fate mapping of MTJ nuclei. The system could also be repurposed for whole-mount or live imaging of the MTJ. We further show that MTJ-AAV can efficiently target muscles throughout the body following systemic administration. Given that different muscle groups exhibit distinct physiological properties and disease susceptibilities (*38*), this platform should facilitate studies of muscle domains in less explored muscles.

Moreover, the same strategy could be extended to other muscle domains, such as the neuromuscular junction, and further refined by combining domain-specific and fiber type–specific promoters to selectively target MTJ or NMJ nuclei associated with particular muscle fiber types. More broadly, this approach may also be applicable to other syncytial systems. For instance, distinct nuclear subtypes have been described in placental syncytiotrophoblasts by snRNA-seq (*39*), suggesting that similar approaches could illuminate nuclear subtype–specific regulation in these contexts as well. Nonetheless, such applications must be pursued with caution, as biomolecular diffusion can confound specificity, as demonstrated by our comparisons of NLS-sfGFP versus H2B-GFP and Rpl22-HA versus GFP-L10a. With careful validation, however, MTJ-AAV and related strategies promise to become powerful tools for dissecting the spatial logic of gene regulation in syncytial cells.

## MATERIALS AND METHODS

### Animals

WT C57BL/6N mice were purchased from Charles River. *Rosa26-LSL-H2B-GFP* mice were described previously (*6*). RiboTag mice were obtained from the Jackson Laboratory (#029977). Mice were housed under standard conditions: constant ambient temperature (23°C), humidity (56%), and a 12-hour light/dark cycle (lights on at 6:00 a.m., lights off at 6:00 p.m.). Animals were euthanized by gradual CO_2_ inhalation (up to 100%) over a 3-min period. All procedures were approved by the institutional animal ethics committee of IGBMC (Comite d’Ethique). Animal health and welfare were continuously monitored by trained staff and veterinarians to minimize suffering.

### AAV construction and generation

*Tigd4* promoter (3.3 kb) was synthesized by GenScript and cloned into pAAV plasmid using pAAV-MCS2 (Addgene) as the backbone. Recombinant AAVs (rAAVs) were generated by a triple transfection of HEK293T/17 cell line using polyethylenimine (PEI) transfection reagent and the following three plasmids: the expression plasmids (pAAV), the pMyoAAV4A encoding Rep and Cap genes, and the pHelper (Agilent) encoding the adenovirus helper functions. After 48 hours of transfection, rAAV vectors were harvested from cell lysate and treated with Benzonase (Merck) at 100 U/ml. They were further purified by gradient ultracentrifugation with iodixanol (Optiprep density gradient medium) followed by dialysis and concentration against Dulbecco’s phosphate-buffered saline using centrifugal filters (Amicon Ultra-15 Centrifugal Filter Devices 100K, Millipore). Viral titers were quantified by real-time PCR using the LightCycler480 SYBR Green I Master (Roche) and primers targeting respective insert sequences (e.g., sfGFP). Titers are expressed as genome copies per milliliter. The pMyoAAV4A plasmid was self-constructed by IGBMC’s molecular biology platform (*40*), benchmarking the published construct (*21*).

### AAV injection

Mice (10 to 12 weeks old) were anesthetized via intraperitoneal injection of a ketamine/xylazine mixture composed of 10% ketamine (100 mg/ml), 5% xylazine (20 mg/ml), and 85% sterile NaCl, administered at a total dose of 100 μl per 10 g of body weight. Anesthesia depth was verified by the absence of pedal reflex before proceeding with the injections.

For intramuscular injection, the skin over the target muscle was disinfected with 70% ethanol under anesthesia. MyoAAV4a vectors carrying different constructs were injected intramuscularly into the TA muscle using a 30-gauge needle. Each construct was administered at its respective final viral genome (vg) dose per muscle: 5 × 10^10^ to 10^11^ vg, depending on the viral concentration and experimental design. The total injection volume per muscle was adjusted with sterile NaCl to ensure a constant final volume of 20 μl across all conditions. Contralateral muscles received vehicle or a control vector where applicable. Muscles were collected at 1-month postinjection for molecular and histological analyses.

For systemic administration, anesthetized mice were placed in a prone position, and MyoAAV4a particles were delivered via retro-orbital sinus injection using a 30-gauge insulin syringe. A total volume of 100 μl corresponding to 1.5 × 10^13^ vg per mouse was injected slowly over approximately 15 s to minimize reflux. Animals were monitored until full recovery and returned to their cages. Tissue collection was performed at 2 months postinjection.

### Ribosome immunoprecipitation and sequencing

Ten-week-old homozygous male RiboTag mice were intramuscularly injected with CK8- or MTJ-AAV carrying Cre recombinase. TA muscles were harvested 1 month later, snap-frozen, and stored at −80°C until processing.

Frozen tissues were thawed on ice and transferred to homogenization tubes containing silica beads (Precellys P000918-LYSK1-A). For the MTJ-AAV condition, lysates were pooled from four injected muscles from different mice. For the CK8-AAV condition, lysates were pooled from two injected muscles and two uninjected WT muscles to normalize total lysate concentration between conditions, as this might affect HA-based immunoprecipitation efficiency and purity.

Each sample was homogenized in 1 ml of lysis buffer containing 50 mM tris-HCl (pH 7.5), 100 mM KCl, 12 mM MgCl_2_, 1% NP-40, 1 mM dithiothreitol (DTT), RNAsin (200 U/ml; Promega), heparin (1 mg/ml; Sigma-Aldrich), cycloheximide (100 μg/ml; Sigma-Aldrich), and protease inhibitor cocktail (Roche). DTT, RNAsin, heparin, cycloheximide, and protease inhibitor were added freshly before use. Tissues were minced with sterile scissors and incubated on ice for 20 min before homogenization using the Precellys Evolution system (5000 rpm, 25 s, twice, with 10-min intervals on ice). Lysates were clarified by centrifugation at 10,000*g* for 20 min at 4°C, and the supernatants were transferred to fresh tubes.

Protein concentration was determined via Bradford assay (Bio-Rad). Approximately 3 mg of lysate was incubated with 5 μl of HA antibody (Covance, clone 12CA5) for 4 hours at 4°C with gentle rotation. Meanwhile, magnetic Protein A/G beads (Pierce) were equilibrated in lysis buffer. Forty microliters of beads was added to each sample, followed by overnight incubation at 4°C with rotation.

Beads were washed three times with high-salt buffer (same as lysis buffer, but with 300 mM KCl and 0.5 mM DTT), each wash involving 5 min of rotation. After the final wash, beads were resuspended in 800 μl of high-salt buffer and transferred to a new tube. RNA was extracted by adding 350 μl of RLT buffer (Qiagen RNeasy kit) and following the manufacturer’s protocol, including DNase treatment. Purified RNA was stored at −80°C until further use.

Library preparation was performed at the GenomEast platform at the Institute of Genetics and Molecular and Cellular Biology using Illumina Stranded Total RNA Prep Ligation with Ribo-Zero Plus (Reference Guide - PN 1000000124514). Total RNA-seq libraries were generated from 50 ng of total RNA using the Illumina Stranded Total RNA Prep, Ligation with Ribo-Zero Plus kit and IDT for Illumina RNA UD Indexes, Ligation (Illumina, San Diego, USA), according to the manufacturer’s instructions. DNA library were amplified using 14 cycles of PCR. Surplus PCR primers were further removed by two successive purifications using SPRIselect beads (Beckman-Coulter, Villepinte, France). The final libraries were checked for quality and quantified using a Bioanalyzer 2100 system (Agilent technologies, Les Ulis, France). Libraries were sequenced on an Illumina NextSeq 2000 sequencer as paired-end 50 base reads. Image analysis and base calling were performed using RTA version 2.7.7 and BCL Convert version 3.8.4.

### Endurance exercise

Ten-week-old homozygous male RiboTag mice were intramuscularly injected with CK8-Cre-NLS or Tigd4-Cre-NLS AAV. One month postinjection, the mice underwent an endurance exercise regimen using an animal treadmill (LE8710, Panlab, Harvard Apparatus, Spain). To ensure proper acclimatization, the mice were introduced to the treadmill environment over a period of 3 days before the start of the experimental protocol. During this familiarization phase, the treadmill operated at a low speed of 10 to 12 cm/s for 15 min each day. The exercise training protocol began at 12 cm/s for 15 min, increased to 20 cm/s for 50 min, and finished with a 5-min cooldown at 10 cm/s. All exercise sessions were consistently conducted at the same time of day for 4 weeks (5 days consecutively and 2 days’ rest).

### FACS isolation and low-input RNA-seq

TA muscles injected with CK8-NLS-sfGFP or Tigd4-NLS-sfGFP AAVs were collected, snap-frozen, and stored at −80°C until further use. The TA muscles were minced and incubated in 300 μl of cold hypotonic buffer [250 mM sucrose, 10 mM KCl, 10 mM MgCl_2_, 10 mM tris-HCl, pH 8, 25 mM Hepes, pH 8, 0.3% Triton X-100, 0.2 mM PMSF, 0.1 mM DTT, and RNase inhibitor (0.2 U/μl)] for 5 min. Samples were then transferred to 2 ml of “Tissue homogenizing CKMix” (Bertin Technologies) with an additional 700 μl of hypotonic buffer. Following a further 15-min incubation, the samples were then homogenized with the “Precellys 24 tissue homogenizer” (Bertin Technologies) for 25 s at 5000 rpm. The homogenized samples were then passed through a 100-μm filter (Sysmex) followed by a 20-μm filter (Sysmex) before the nuclei were pelleted by centrifugation at 400*g* for 10 min at 4°C. The pellets were then washed and resuspended in a washing buffer [2% bovine serum albumin (BSA) in phosphate-buffered saline (PBS) + RNase inhibitor (0.2 U/μl)]. The centrifugation and wash steps were then repeated before the homogenized samples were passed through a “5-ml polystyrene round-bottom tube with cell-strainer cap” (Corning). DAPI was then added to the resuspended nuclei at a final concentration of 200 mM. The isolated nuclei were sorted with FACs Aria Fusion 2022, with BD FACSDiva and FlowJo (v10) software, to sort out the DAPI and GFP^+^ nuclei. Approximately 1000 nuclei were collected from each sample. The sorted nuclei were immediately collected in 11.5 μl of CDS sorting solution, SMART-seq mRNA LP (with UMIs) recipe (Takara), briefly spun down, then flash-frozen on dry ice. The frozen isolated nuclei were then stored at −80°C until further use.

Library preparation was performed at the GenomEast platform at the Institute of Genetics and Molecular and Cellular Biology using Takara Bio USA Inc., SMART-seq mRNA User Manual - PN 062223 + Illumina Nextera XT DNA Library Prep Kit (Reference Guide - PN 15031942). Full-length cDNA were generated from 100 to 2000 nuclei using SMART-seq mRNA (Takara Bio Europe, Saint Germain en Laye, France) according to the manufacturer’s instructions with 11 cycles of PCR for cDNA amplification by Seq-Amp DNA polymerase. The entire volume of each preamplified cDNA was then used as input for Tn5 transposon tagmentation followed by 12 cycles of library amplification using Nextera XT DNA Library Preparation Kit and IDT for Illumina DNA/RNA UD Indexes, Tagmentation (Illumina, San Diego, USA). Following purification with SPRIselect beads (Beckman-Coulter, Villepinte, France), the size and concentration of libraries were assessed by capillary electrophoresis (Bioanalyzer 2100 system, Agilent Technologies, Les Ulis, France). Full-length cDNA was generated from 100 to 2000 nuclei using SMART-seq mRNA Kit (Takara Bio Europe, Saint Germain en Laye, France) according to the manufacturer’s instructions with 11 cycles of PCR amplification by Seq-Amp polymerase. Totality of preamplified cDNA was then used as input for Tn5 transposon tagmentation followed by 12 cycles of library amplification using Nextera XT DNA Library Preparation Kit and IDT for Illumina DNA/RNA UD Indexes (Illumina, San Diego, USA). Following purification with SPRIselect beads (Beckman-Coulter, Villepinte, France), the size and concentration of libraries were assessed using a Bioanalyzer 2100 system (Agilent Technologies, Les Ulis, France). Libraries were sequenced on an Illumina NextSeq 2000 sequencer as paired-end 50 base reads. Image analysis and base calling were performed using RTA version 2.7.7 and BCL Convert version 3.8.4.

### Bioinformatic analyses of RNA-seq datasets

All sequencing was performed on an Illumina NextSeq 2000 platform in a 2× 50-bp paired-end configuration. For RiboTag-seq, sequencing data were processed using a PiGx-RNA-seq (30277498) pipeline. In short, the data were mapped onto the GRCm39/mm11 version of the mouse transcriptome (downloaded from the ENSEMBL database 29155950) using SALMON (28263959). The quantified data were processed using tximport (26925227), and the differential expression analysis was done using DESeq2 (25516281). Genes with less than five reads in all biological replicates of one condition were filtered out before the analysis. Two groups of differentially expressed genes were defined—a relaxed set containing genes with an absolute log2 fold change of 0.5, and a stringent set containing games with an absolute log2 fold change of 1. The fold change was deemed significant if the adjusted *P* value was less than 0.05 (Benjamini-Hochberg corrected).

For bulk RNA-seq, reads were preprocessed with cutadapt 4.2 to remove adaptor sequences, poly(A) tails, and low-quality bases. Reads shorter than 40 bp were discarded. The remaining reads were mapped to *Mus musculus* rRNA sequences using bowtie2 2.3.5 (*41*) and reads aligning to these sequences were removed. The filtered reads were then aligned to the *M. musculus* reference genome (GRCm39 assembly) using STAR 2.7.10b (*42*). Gene-level quantification was performed with HTSeq-count 1.99.2 (*43*) in “union” mode, using Ensembl 111 annotations. Differential gene expression analysis was carried out with the DESeq2 1.34.0 (*44*) R/Bioconductor package using default parameters. *P* values were adjusted for multiple testing with the Benjamini-Hochberg method. GO and pathway reactome analyses were performed using the https://maayanlab.cloud/Enrichr/ website.

### Analyses of the snATAC-seq dataset and motif enrichment

snATAC-seq libraries were processed using Signac v1.10.0 and Seurat v4.3.0 in R. Raw peak files were imported and converted to GRanges objects for genomic coordinate standardization. Cell-type–specific open chromatin peaks were identified for MTJ nucleus through Seurat object integration with parallel transcriptomic data (GEX), using clustering and differential accessibility analysis to isolate peaks enriched in MTJ-specific clusters. From the total accessible chromatin landscape, MTJ-specific peaks were extracted by filtering for regions showing significant accessibility exclusively in MTJ nucleus relative to other muscle nucleus populations. Peak coordinates were standardized using StringToGRanges coordinate conversion (mm10 genome build). Selected peaks were then used as input for downstream transcription factor binding site (TFBS) discovery via motif scanning with JASPAR 2020 vertebrate PWMs.

TFBS enrichment analysis was performed on MTJ-specific open chromatin regions using a computational motif-scanning approach. MTJ-specific accessible chromatin peaks were identified from ATAC-seq data and used as input for systematic motif discovery. Vertebrate transcription factor position weight matrices (PWMs) were obtained from the JASPAR 2020 database using TFBSTools (v1.38.0). All vertebrate PWMs with nonredundant versions were retrieved, encompassing 746 motif models representing diverse transcription factor families. Genomic sequences corresponding to each peak region were extracted from the mouse reference genome (mm10) using BSgenome.Mmusculus.UCSC.mm10 (v1.4.3). Motif scanning was performed with motifmatchr (v1.22.0) using the matchMotifs() function, which implements a log-odds scoring algorithm based on PWM models. For each peak-motif pair, a match score was computed by sliding the PWM across both DNA strands. Binding sites exceeding the default log-odds threshold (typically *P* < 10^−4^) were classified as putative TFBS occurrences.

Results were integrated into a Seurat object (v4.3.0) using the Signac framework (v1.10.0) for chromatin accessibility data. A binary motif occurrence matrix was constructed with dimensions *n* peaks × *m* motifs, where each entry indicates the presence (1) or absence (0) of a motif within a given peak. This matrix was stored as a ChromatinAssay object within the Seurat framework, enabling downstream integration with single-cell chromatin accessibility profiles when applicable.

Global motif enrichment was calculated as the proportion of peaks containing each motif

Enrichment (%) = (Number of peaks with motif/Total peaks) × 100

Motifs were ranked by frequency of occurrence across all analyzed peaks compared to random occurrences. The top-ranking motifs representing the most enriched TFBSs in MTJ-specific open chromatin were identified for downstream validation and functional interpretation.

MTJ-specific accessible chromatin peaks were functionally annotated using ChIPseeker v1.36.0 with the mouse genome annotation database (TxDb.Mmusculus.UCSC.mm10.knownGene and org.Mm.eg.db). Peak coordinates were mapped to their nearest genomic features, including promoters (±3 kb from transcription start sites), gene bodies, introns, and intergenic regions, using annotatePeak(). The distribution of peaks across genomic features was visualized with annotation pie charts to assess regulatory landscape composition. Gene symbols associated with each peak were extracted, and genes with multiple associated peaks (≥150 peaks per gene) were identified as high-confidence MTJ regulatory targets. This threshold-based filtering enriched for genes with extensive regulatory architecture characteristic of master regulators and structural genes defining MTJ identity.

All analyses were performed in R (v4.3.1). The complete analysis pipeline, including motif occurrence matrices and enrichment statistics, was saved for reproducibility and downstream chromVAR-based activity inference.

### snRNA-seq and analyses

Library preparation was performed at the GenomEast platform at the Institute of Genetics and Molecular and Cellular Biology using Chromium Next GEM Single Cell 3 prime Reagent Kits User guide v3.1 (Dual index) - PN CG000315. Single-nuclei libraries were generated using the Single Cell 3′ Reagent Kit v3.1 chemistry following the guidelines of the 10X Genomics user manual (10X Genomics, Leiden, The Netherlands). Single-nuclei suspensions were processed on the 10X Genomics Chromium iX targeting 10,000 captured nuclei per sample. Full-length cDNA amplification was carried out using 11 cycles of PCR. Dual index library construction was performed with 14 cycles during sample index PCR. Final library quantification and quality control were completed using Bioanalyzer 2100 (Agilent Technologies, Les Ulis, France). Libraries were sequenced on an Illumina NextSeq 2000 sequencer as paired-end 28 + 85 base reads. Image analysis and base calling were performed using RTA version 4.12.2 and BCL Convert version 4.2.7.

Because the two transgenes share a 3′ backbone that multimaps against each other in a combined reference, condition-specific references were built: reads from control legs were aligned to a reference comprising the mouse genome (mm39, Ensembl release 113) augmented with the Cre transgene contig, and reads from KLF2-DN legs to mm39 augmented with the KLF2-DN transgene contig. Each transgene was added as a single-exon contig so that transduced nuclei could be scored by transgene UMIs. Reads were aligned and quantified with STARsolo (STAR v2.7.10b) using “--soloType CB_UMI_Simple,” “--soloFeatures GeneFull” (intron-inclusive, pre-mRNA counting appropriate for single-nucleus data), “--soloUMIlen 12,” “--sjdbOverhang 84,” and the GEM-X v4 barcode whitelist (“3M-3pgex-may-2023.txt”). Nuclei were called from the knee of the barcode-rank distribution (“--soloCellFilter CellRanger2.2 10000 0.99 10”). Per-library filtered nucleus × gene count matrices from the GeneFull quantification were used for all downstream analysis.

Analysis was carried out in R 4.5.0 (Bioconductor 3.21) with Seurat v5.5.0. Each library was loaded independently. Nuclei were retained if they expressed 200 to 7000 genes and had less than 10% mitochondrial (“^mt-”) counts. Retained nuclei were log-normalized (“NormalizeData,” default scale factor 10,000), and the 3000 most variable genes were selected (“FindVariableFeatures,” variance-stabilizing transform) and scaled (“ScaleData”). Principal component analysis was computed on the scaled data (“RunPCA,” 50 components). A shared-nearest-neighbor graph was built on the first 30 principal components (“FindNeighbors”), Louvain clustering was performed at resolution 0.6 (“FindClusters”), and a UMAP embedding was computed on the same 30 components (“RunUMAP”). Default Seurat random seeds were used throughout, so results are deterministic given identical input.

Putative doublets were identified per library with scDblFinder (v1.22.0) in cluster-aware mode on the raw counts, run in a single process (“BiocParallel::SerialParam”). Ambient-RNA contamination was estimated per nucleus with SoupX (v1.6.2) using the STARsolo raw (unfiltered) droplet matrix and the per-library clusters, with automatic contamination estimation (“autoEstCont,” falling back to a fixed 0.1 fraction where estimation failed).

For joint analyses, the eight per-library objects were merged, renormalized and rescaled, and integrated across libraries using Seurat’s reciprocal-PCA method (“IntegrateLayers,” “method = RPCAIntegration,” “orig.reduction = "pca",” and “new.reduction = "integrated.rpca"”). Harmony/batchelor-based integration was not used as those packages were absent from the analysis environment; RPCA is native to Seurat and requires no additional dependency. Neighbors, Louvain clustering (resolution 0.6), and UMAP were recomputed on the first 30 dimensions of the integrated embedding. To avoid exporting the merged object to parallel workers, integration was run under a sequential future plan with an enlarged global-size limit. A per-mouse integration (the two legs of each mouse integrated independently by RPCA) was performed analogously as an additional definition of the MTJ population.

The MTJ signature was derived from the Kim *et al.* and Petrany *et al. *(*6**,* *7*) mouse muscle snRNA-seq atlas: The atlas MTJ cluster (highest fraction of Col22a1-positive nuclei) was compared to all other clusters (“FindMarkers,” one-vs-rest, positive markers only, “logfc.threshold = 0.25”), and the top 200 genes by average log2 fold change with adjusted *P* < 0.05 were retained. Per-nucleus signature scores were computed with “AddModuleScore” (control set of 50 background genes).

Using a threshold-independent check, the MTJ signature score was min-max scaled to [0, 1] within each library (over scDblFinder singlets), and the fraction of nuclei exceeding a range of score thresholds (0.30 to 0.90) was compared between conditions with the same paired test. Given *n* = 4, these tests are underpowered and were interpreted as trend indicators rather than calibrated significance.

Per-nucleus transgene load (“transduction”) was quantified as the UMI count of the library’s own transgene contig (Cre in control legs, KLF2-DN in DN legs); nuclei with ≥1 transgene UMI were considered transgene-positive. Because the integrated cassette is intronless and 3′ snRNA capture is biased toward polyadenylated, intron-containing transcripts, transgene capture is sparse and transgene-negative nuclei cannot be assumed untransduced; transgene status was therefore used as a low-sensitivity presence marker only. Genes differentially expressed between transgene-positive and transgene-negative nuclei were identified within each condition (pooling the four same-condition legs) with “FindMarkers” (Wilcoxon rank-sum test, “logfc.threshold = 0.25,” “min.pct = 0.1,” Bonferroni-adjusted *P* values); median detected-gene counts of the two groups were reported alongside to flag the capture-depth confound.

### Histology and imaging

For histological analysis, samples were rapidly embedded in Cryomatrix (Epredia) in cryomolds then snap-frozen in isopentane precooled in liquid nitrogen for approximately 15 s until the cryomatrix solidified completely. Samples embedded in cryomatrix were equilibrated to cryostat temperature (−20°C) before sectioning. Transverse cryosections were cut at 10 μm thickness using a Leica CM3050S cryostat (Leica Microsystems, Germany) and collected on Superfrost Plus adhesion microscope slides (Epredia). Sections were then stored at −20°C for 30 min then stored at −80°C until further use.

For RNAscope, the tissue sections were fixed with 4% paraformaldehyde (PFA) in PBS for 15 min at 4°C. After two times washing with PBS, the sections were serially dehydrated through increasing ethanol (50, 70, and 100%) 5 min each. Subsequently, RNAscope was performed according to the manufacturer’s guideline using the RNAscope Multiplex Fluorescent Reagent Kit v2 (Bio-Techne 323100). Proteinase IV was used for our procedure. The following RNAscope probes were used for our study: *Col22a1* (590911-C2), *Tigd4* (598761), *Ttn* (483031), *GFP* (409011), and *Engrailed* (newly designed for this study). After all RNAscope procedures, samples were counterstained with DAPI and mounted using Prolong Gold Antifade (Thermo Fisher Scientific). When combined with antibody staining, tissue sections were processed to blocking buffer (see below) after the last wash and proceeded to regular immunohistochemistry.

For immunofluorescence staining, transverse cryosections were fixed in 4% PFA for 10 min at room temperature (RT), followed by three washes in PBT (PBS containing 0.1% Tween-20) for 5 min each. Permeabilization was performed in PBX (PBS containing 0.5% Triton X-100) for 6 min, after which sections were rinsed in PBS for 5 min. Nonspecific binding was blocked by incubating the sections in blocking buffer (PBS containing 5% BSA, 3% horse serum, and 0.1% Triton X-100) for 1 hour at RT. Sections were then incubated with primary antibody diluted in blocking buffer overnight at 4°C in a humidified chamber. The following day, slides were washed three times in PBT for 10 min each, then incubated with fluorophore-conjugated secondary antibody diluted in blocking buffer for 1 hour at RT. DAPI (1 μg/ml) was included in the secondary incubation step for nuclear counterstaining. Last, sections were washed three times in PBT for 10 min each in the dark, mounted with ProLong Gold Antifade, and stored at 4°C until imaging. The following antibodies were used in this study: GFP (Aves Labs; 1:500), Klf2 (Cell Signaling, 1:500), Dystrophin (Abcam, 1:250), and Col22a1 (Gift from Manuel Koch, 1:1000).

Images were acquired on a Leica confocal at the IGBMC imaging core facility. A 20× oil-immersion objective was used. Fluorescent signals were sequentially detected using appropriate laser lines and emission filters for DAPI, Opal 520, Opal 570, and Opal 690 (Opal chemicals were from Akoya Biosciences) to avoid channel bleed-through. Laser power, gain, and offset were optimized and maintained constant across samples. Imaging was performed using the tile scan mode to capture large tissue areas, with a frame size of 520 × 520 pixels and processed using ImageJ.

### Fluorescence intensity quantification

All immunostaining and RNAscope experiments for quantification were performed simultaneously. Images were acquired using identical microscope settings (laser power, detector gain, exposure time, and objective) for all samples within each experiment. Image analysis was performed using ImageJ/Fiji. A fixed-size region of interest (ROI) was placed over the tip of the myofiber at the MTJ, and an identical ROI was placed over the adjacent body of the same fiber, which served as the reference region. Mean fluorescence intensity was measured for both ROIs and expressed in arbitrary units. Local enrichment at the MTJ was calculated as the difference between the mean fluorescence intensity of the tip ROI and that of the adjacent body ROI. The same ROI size and analysis parameters were applied to all images.

For tissue sections, fluorescence at the MTJ was analyzed separately from single fibers. MTJ regions were manually outlined on raw images using anatomical landmarks of the muscle-tendon interface. Because the MTJ is a variable and heterogeneous region, ROI size was not kept constant between samples. Mean fluorescence intensity was measured within each MTJ ROI, providing a size-independent estimate of signal density. Background fluorescence was measured in a signal-free area of the same section and subtracted from MTJ values. The corrected mean intensities were used for statistical analysis.

### Western blotting

TA tissues were lysed as described in the “Ribosome immunoprecipitation and sequencing” section. Lysates were denatured by adding Laemmli buffer and boiling for 10 min. Denatured samples were separated by SDS–polyacrylamide gel electrophoresis (Bio-Rad) and transferred into nitrocellulose membrane (Amersham). Transferred membranes were blocked for 1 hour with 5% skim milk in TBS plus 0.1% Tween-20 (TBST) at RT. Afterward, primary antibodies were incubated in 5% BSA in TBST supplemented with 0.1% sodium azide overnight in a cold room with gentle rocking. After washing three times with TBST (10 min each), membranes were incubated with secondary antibodies (anti-mouse or anti-rabbit HRP; Cell Signaling) diluted in skim milk (1:5000) for 1 hour in RT. After three times washing with TBST, membranes were developed using chemiluminescence (ECL substrate; Pierce) and Amersham ImageQuant 800. For streptavidin-HRP, antibody was incubated for 1 hour in skim milk after blocking. The following antibodies were used in this study: Col22a1 (Abcam; 1:500), β-actin (Cell Signaling; 1:1000), Klf2 (Cell Signaling, 1:500), HA (Covance, 1:2000), and streptavidin-HRP (Sigma-Aldrich, 1:5000).

### Statistical analysis

Graph generation and statistical analyses were performed using GraphPad Prism as described in each figure legend.
