# Supplementary material for: Local translational program at the muscle-tendon junction endows domain identity in muscle syncytia
Source: Sci Adv. 2026 Nov 4;12(45):eaee6166. doi: 10.1126/sciadv.aee6166 (PMC13733890; doi:10.1126/sciadv.aee6166)
Supplement: Supplementary file 1 [file sciadv.aee6166_sm.pdf]

Science Advances
AAAS

Supplementary Materials for

Local translational program at the muscle-tendon junction endows domain identity in muscle syncytia

Nour El Khazen *et al.*

Corresponding author: Minchul Kim, kimm@igbmc.fr

Sci. Adv. **12**, eaee6166 (2026)
DOI: 10.1126/sciadv.aee6166

The PDF file includes:

Figs. S1 to S10
Legends for tables S1 to S3
Legend for movie S1
Legend for file S1

Other Supplementary Material for this manuscript includes the following:

Tables S1 to S3
Movie S1
File S1

3D rednered image of Figure 1C

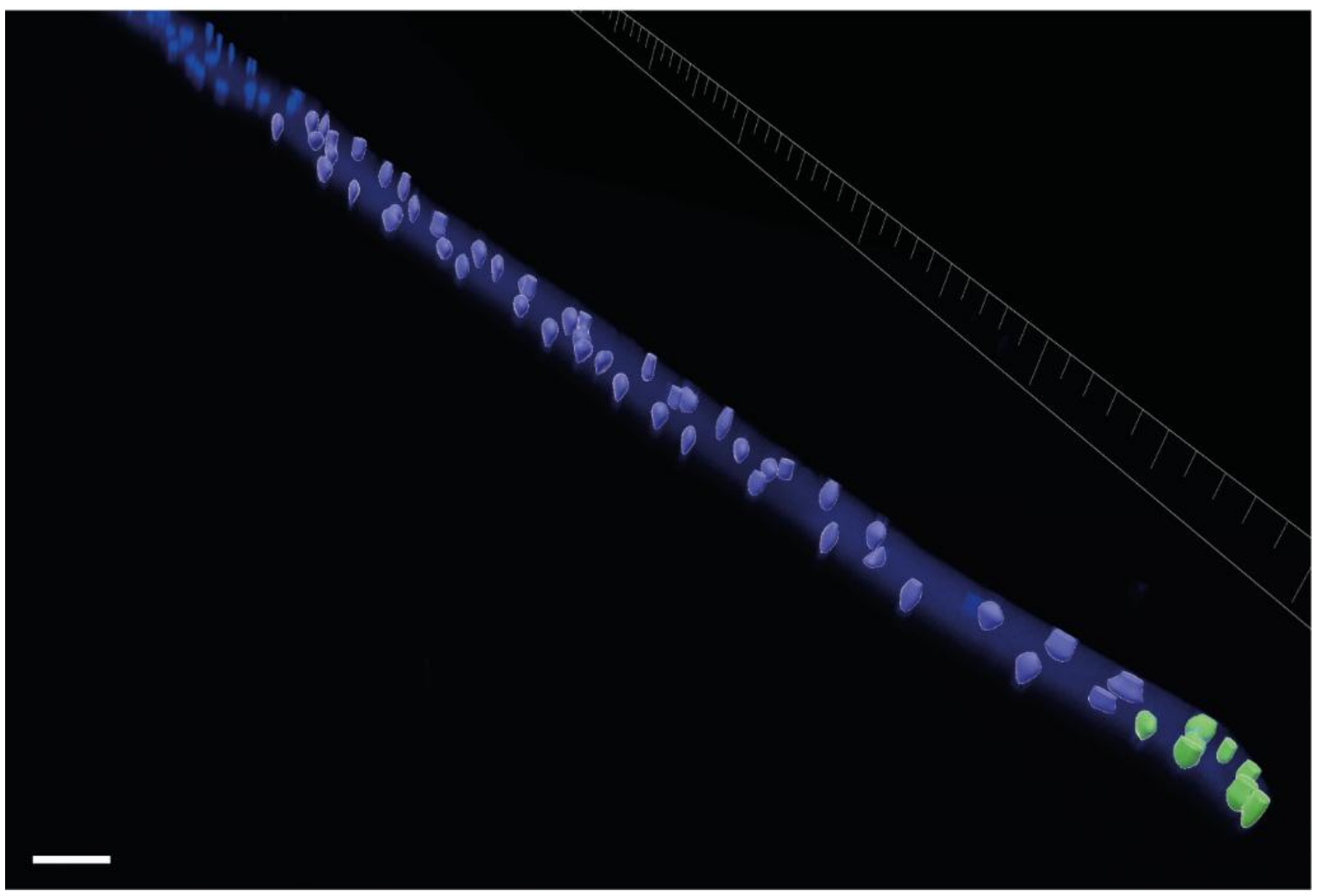

Figure S1. Expression of 4×NLS-sfGFP reporter at the MTJ in isolated EDL myofibers
The myofiber shown in Fig. 1C was subjected to z-stack acquisition followed by 3D rendering. A rotating view of the entire fiber is provided in Supplementary Video 1.

Specificity of MTJ-AAV expressing 4xNLS-sfGFP

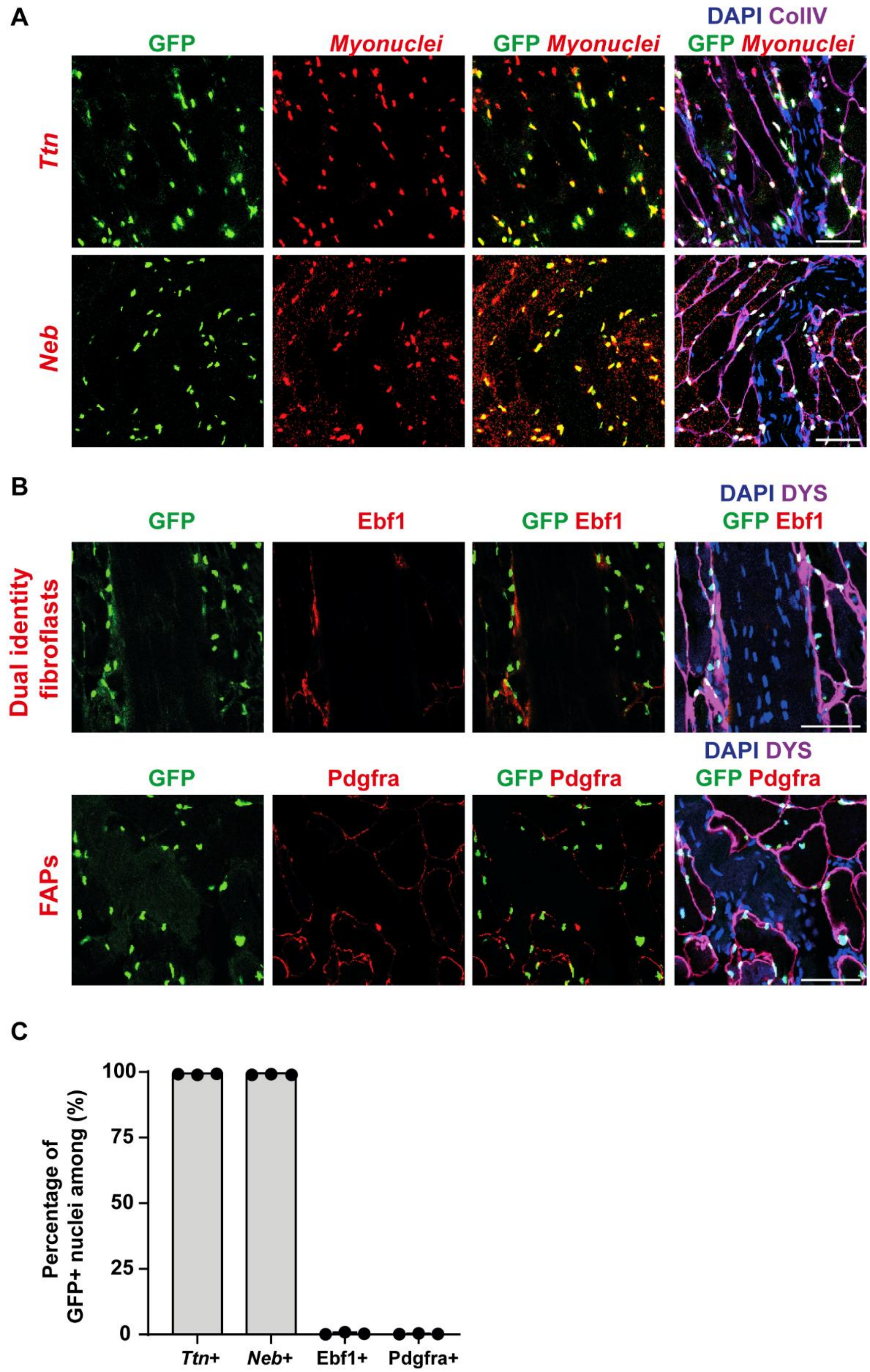

Figure S2. Specificity of the MTJ-AAV system (4xNLS-sfGFP)

(A) RNAscope for *Ttn* or *Neb* combined with GFP and Collagen IV (ColIV) immunostaining in longitudinal TA sections from mice intramuscularly injected with MTJ-AAV carrying the 4×NLS-sfGFP reporter. All GFP-positive nuclei co-express *Ttn* and *Neb*, confirming their myonuclear identity.

(B) Co-immunostaining of GFP with the indicated lineage markers, demonstrating that GFP-positive nuclei do not co-localize with these cell populations.

(C) Quantification of the experiments shown in (A) and (B) (n = 3).

Error bars indicate S.E.M. Scale bars, 50 µm.

Specificity after Cre-mediated recombination at the MTJ to express H2B-GFP

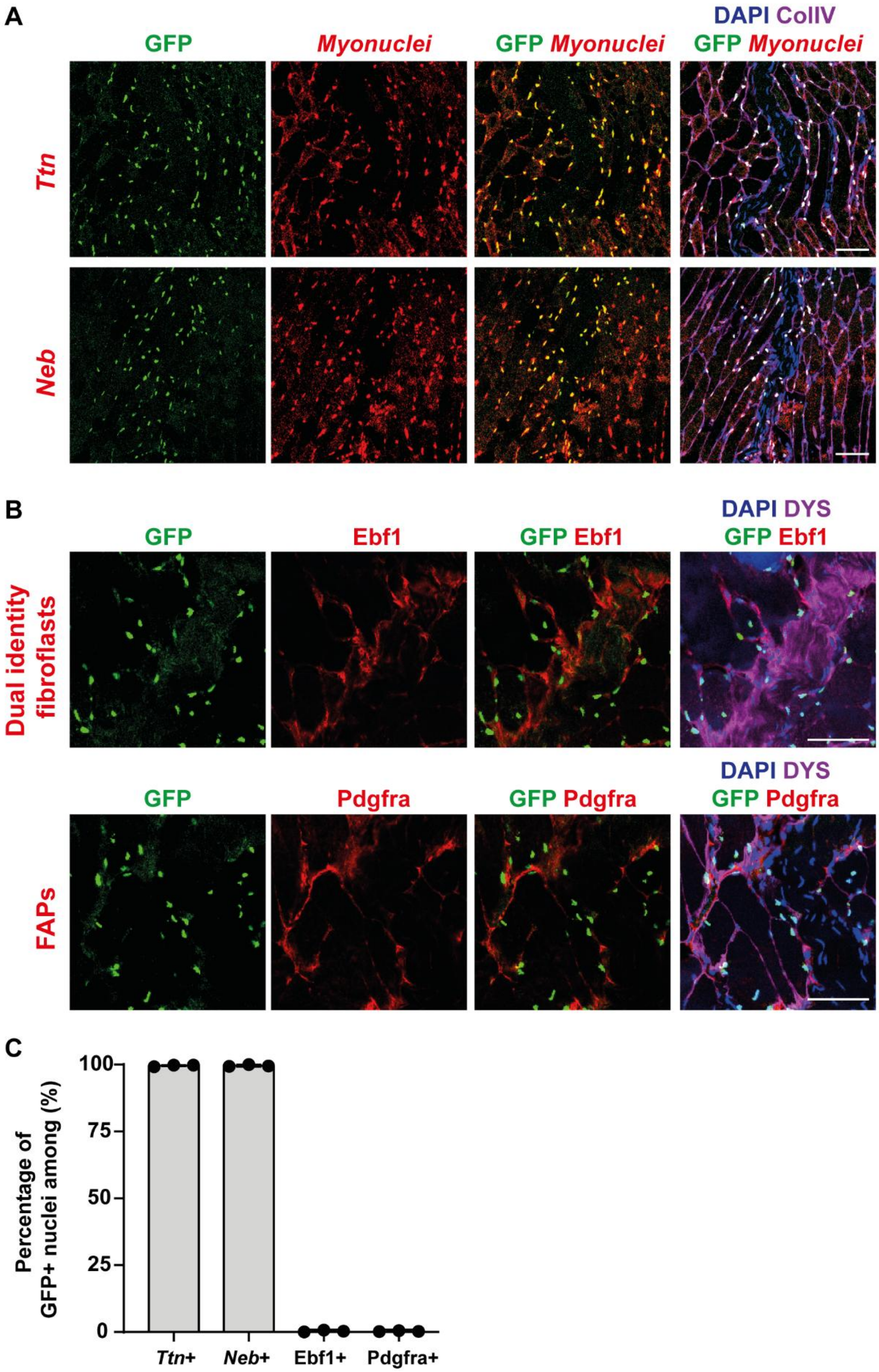

Figure S3. Specificity of the MTJ-AAV system (H2B-GFP)
(A-C) Same analyses as shown in Fig. S2, but performed in *Rosa26-LSL-H2B-GFP* reporter mice following intramuscular injection of MTJ-AAV carrying Cre-NLS (n = 3).
Error bars indicate S.E.M. Scale bars, 50 µm.

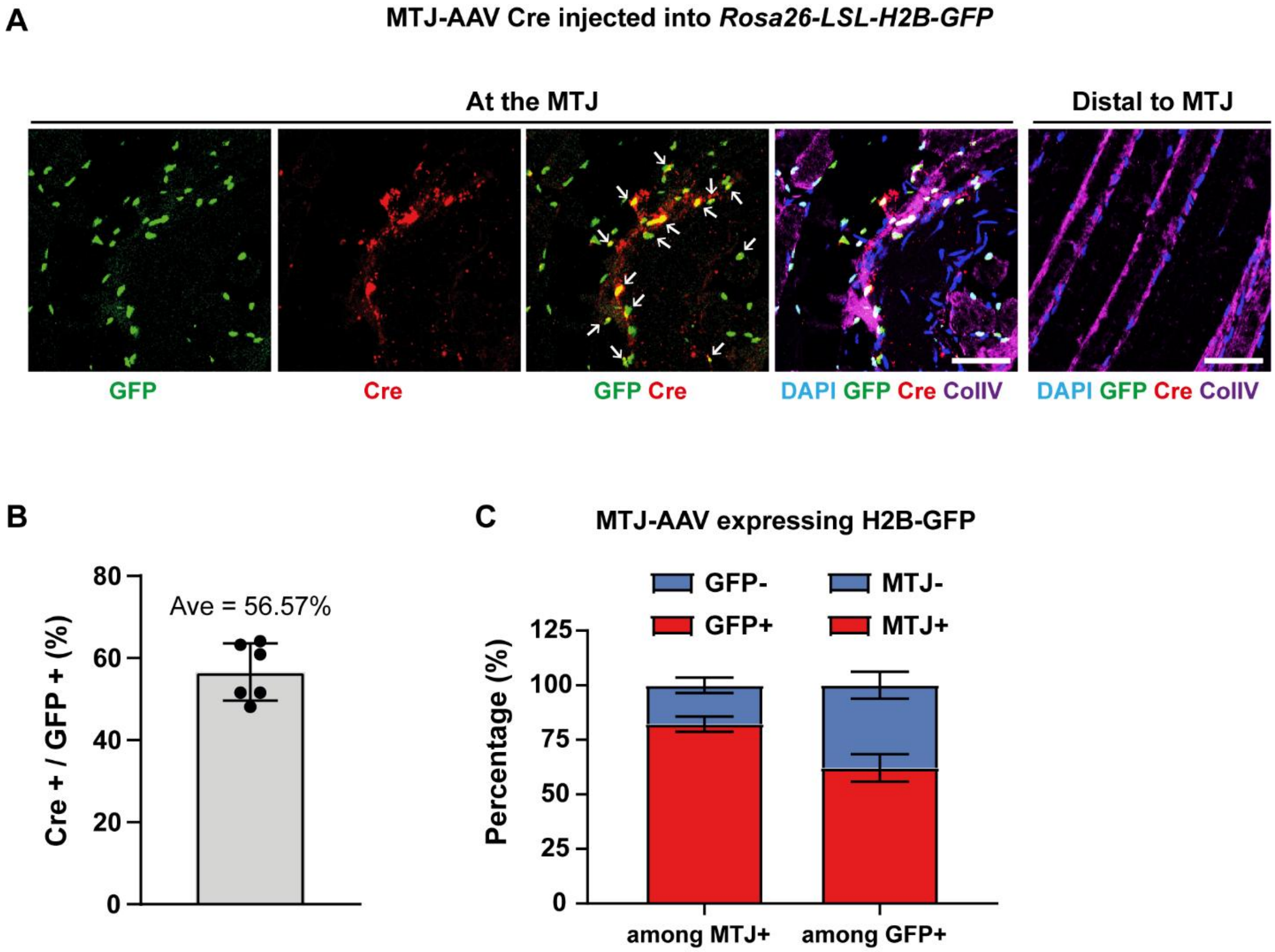

Figure S4. H2B-GFP is more diffusive than 4xNLS-sfGFP

(A) Immunohistochemistry for Cre and GFP in TA muscles of *Rosa26-LSL-H2B-GFP* mice injected with MTJ-AAV-Cre-NLS. Arrows indicate GFP and Cre double positive nuclei. Scale bars, 50 µm.

(B) Quantification of Cre-positive nuclei among GFP-expressing nuclei (n = 6).

(C) Wild-type mice were intramuscularly injected with MTJ-AAV-H2B-GFP, and labeling efficiency and specificity were quantified (n = 3).

Error bars indicate S.E.M.

Ribosome labeing using *Rosa26-LSL-GFP-L10a* line

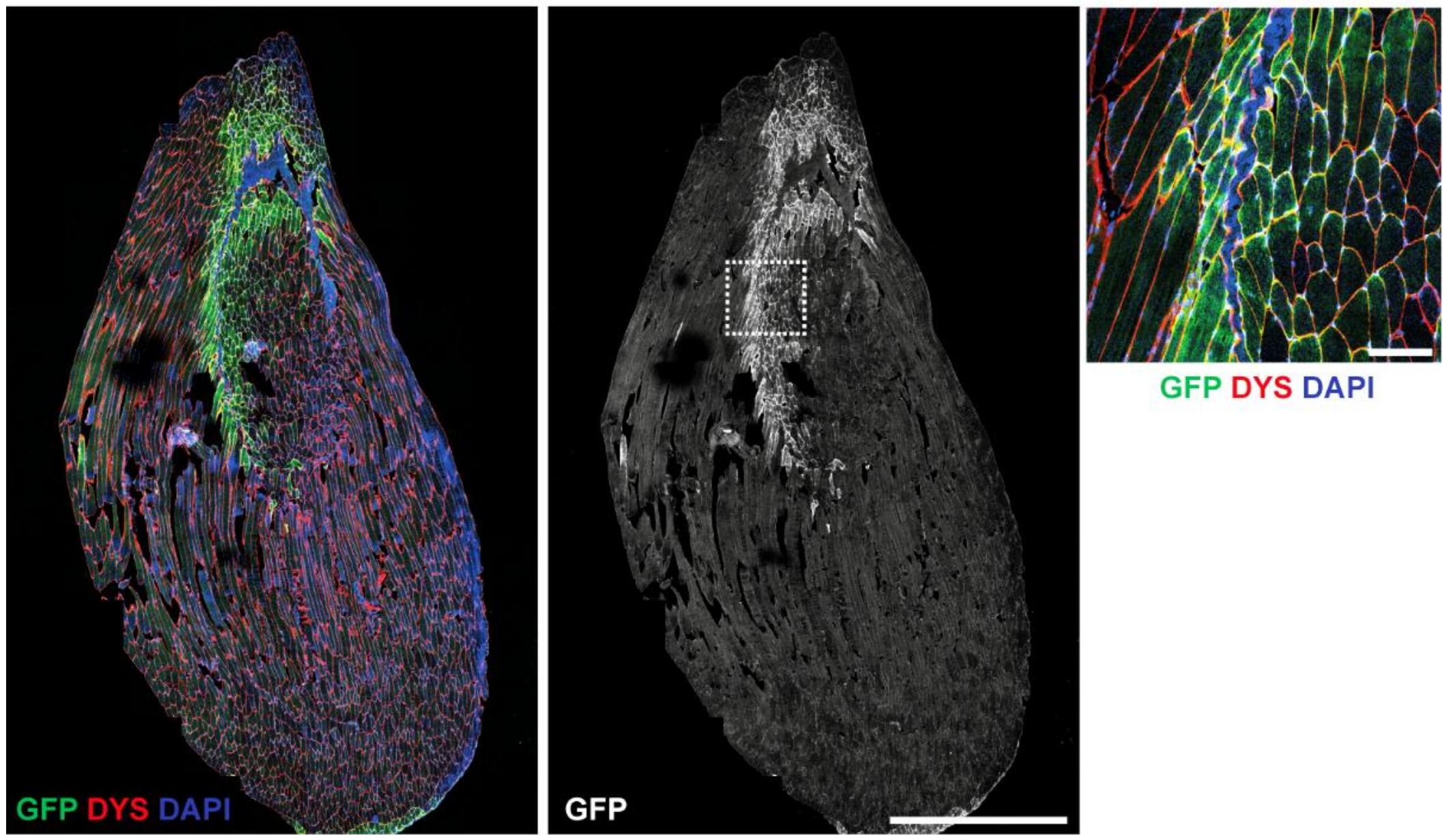

Figure S5. Ribosome tagging using the Ribo-Trap line results in a more diffuse labeling pattern than the RiboTag line

Tile-scan images of TA muscles from *Rosa26-LSL-GFP-L10a* mice injected with MTJ-AAV carrying Cre-NLS. The right panels show higher-magnification views of the boxed regions. Scale bars, 1000 µm for the tile-scan image and 100 µm for the inset image.

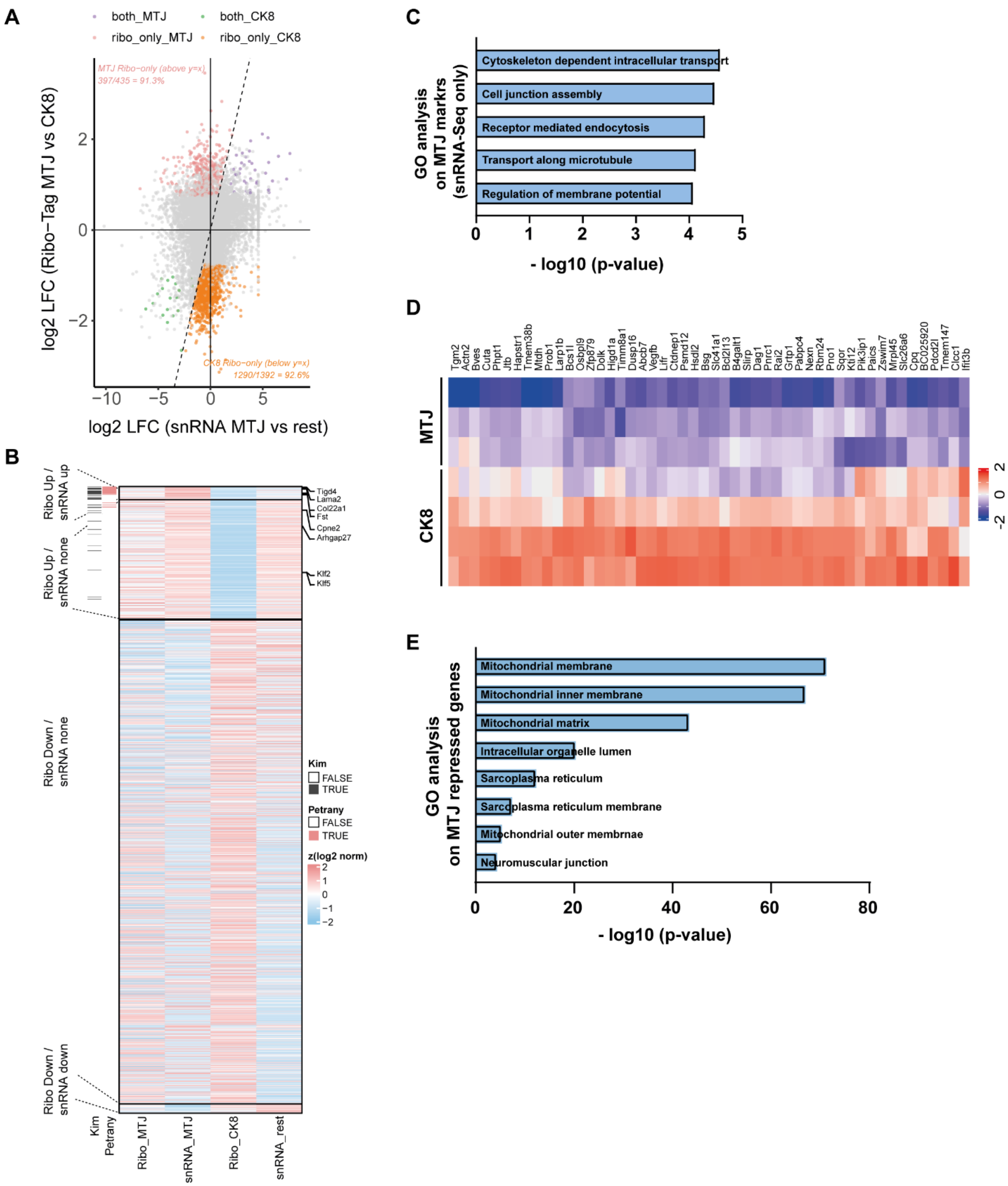
A
both_MTJ
both_CK8
ribo_only_MTJ
ribo_only_CK8
MTJ Ribo-only (above y=x)
397/435 = 91.3%
CK8 Ribo-only (below y=x)
1290/1392 = 92.6%
log2 LFC (Ribo−Tag MTJ vs CK8)
log2 LFC (snRNA MTJ vs rest)
B
Ribo Up / snRNA up
Ribo Up / snRNA none
Ribo Down / snRNA none
Ribo Down / snRNA down
Tigd4
Lama2
Col22a1
Fst
Cpne2
Arhgap27
Klf2
Klf5
Kim
FALSE
TRUE
Petrany
FALSE
TRUE
z(log2 norm)
Kim
Petrany
Ribo_MTJ
snRNA_MTJ
Ribo_CK8
snRNA_rest
C
GO analysis on MTJ markrs (snRNA-Seq only)
Cytoskeleton dependent intracellular transport
Cell junction assembly
Receptor mediated endocytosis
Transport along microtubule
Regulation of membrane potential
- log10 (p-value)
D
Tgm2
Actn2
Bves
Cuta
Phpt1
Jtb
Hapstr1
Tmem38b
Mtdh
Prob1
Larp1b
Bcs1l
Osbpl9
Zfp879
Dlk
Higd1a
Timm8a1
Dusp16
Abcb7
Vegfb
Lifr
Ctdnep1
Psmd12
Hsdl2
Bsg
Slc41a1
Bcl2l13
B4galt1
Slirp
Dag1
Pnrc1
Ral2
Grtp1
Pabpc4
Nexn
Rbm24
Pno1
Sqor
Klf12
Pik3ip1
Paics
Zswim7
Mrpl45
Slc26a6
Cpq
BC025920
Pdcd2l
Tmem147
Clcc1
Ifi3b
MTJ
CK8
E
GO analysis on MTJ repressed genes
Mitochondrial membrane
Mitochondrial inner membrane
Mitochondrial matrix
Intracellular organelle lumen
Sarcoplasma reticulum
Sarcoplasma reticulum membrane
Mitochondrial outer membrnae
Neuromuscular junction
- log10 (p-value)

Figure S6. Analyses related to MTJ translational profiling in sedentary muscles

(A) Scatter plot comparing the RiboTag-Seq and snRNA-seq datasets (Petrany *et al.*). Note the substantial discordance between the translatome and transcriptome, and the four gene categories defined by their differential enrichment patterns.

(B) Heatmap showing genes enriched or depleted at the MTJ at the translatome and/or transcriptome level. 'snRNA none' indicates genes that were not significantly enriched nor depleted at the transcript level. For each gene, its identification as an MTJ transcriptional marker in the two published studies (Kim *et al.* and Petrany *et al.*) is indicated on the left. Note that these previously identified MTJ markers are heavily concentrated in the 'Ribo up / snRNA up' category, indicating that their MTJ enrichment is largely explained by transcription. The complete gene list is provided in Supplementary Table 2.

(C) Gene Ontology (biological process) analysis of genes enriched exclusively at the transcriptome level in the MTJ.

(D) Heatmap showing representative MTJ ribosome-depleted transcripts.

(E) Gene Ontology analysis of translationally depleted genes at the MTJ.

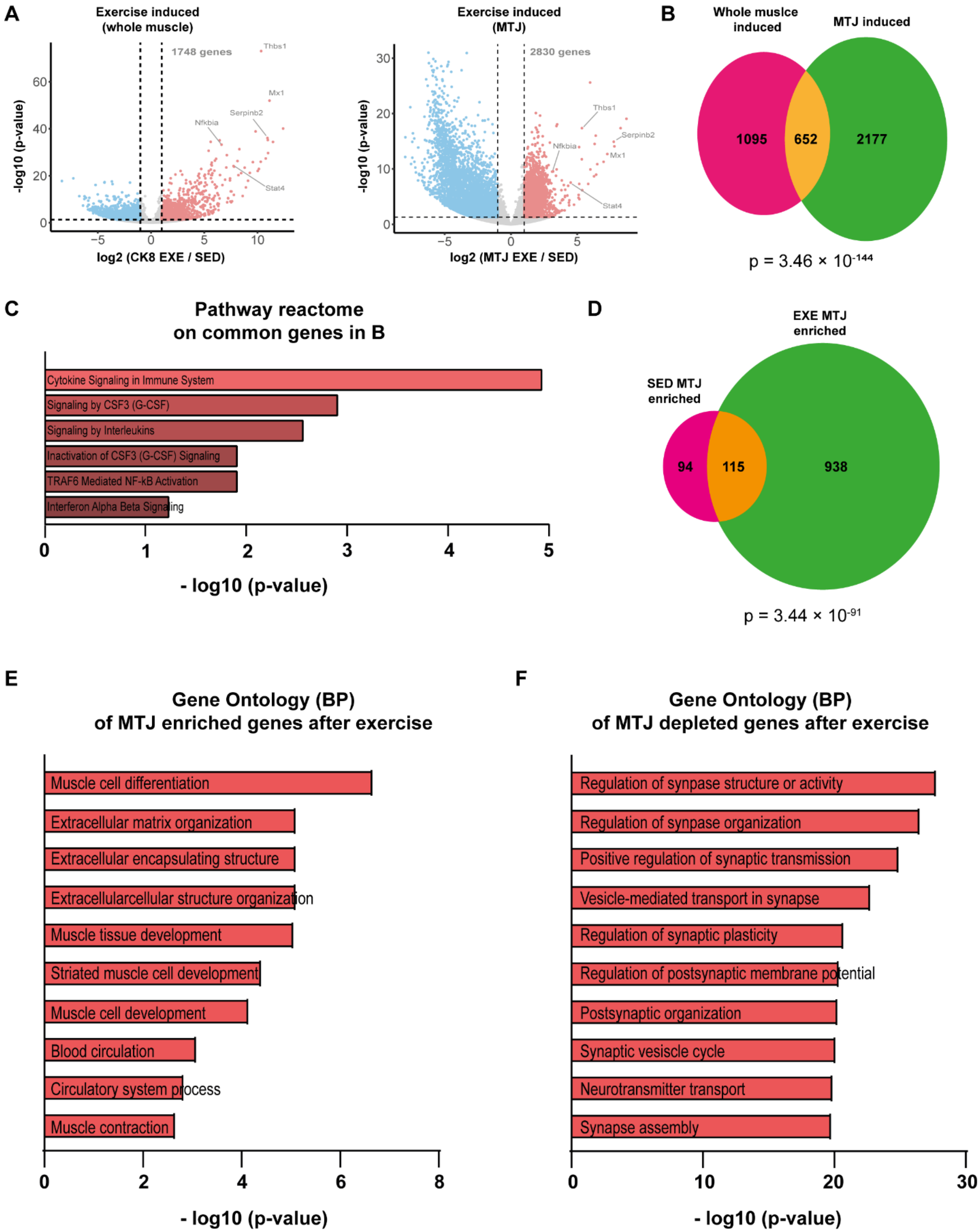

A
Exercise induced (whole muscle)
1748 genes
Thbs1
Mx1
Serpinb2
Nfkbia
Stat4
-log10 (p-value)
log2 (CK8 EXE / SED)
Exercise induced (MTJ)
2830 genes
log2 (MTJ EXE / SED)
B
Whole muslce induced
MTJ induced
1095
652
2177
p = 3.46 × 10-144
C
Pathway reactome on common genes in B
Cytokine Signaling in Immune System
Signaling by CSF3 (G-CSF)
Signaling by Interleukins
Inactivation of CSF3 (G-CSF) Signaling
TRAF6 Mediated NF-kB Activation
Interferon Alpha Beta Signaling
- log10 (p-value)
D
EXE MTJ enriched
SED MTJ enriched
94
115
938
p = 3.44 × 10-91
E
Gene Ontology (BP) of MTJ enriched genes after exercise
Muscle cell differentiation
Extracellular matrix organization
Extracellular encapsulating structure
Extracellularcellular structure organization
Muscle tissue development
Striated muscle cell development
Muscle cell development
Blood circulation
Circulatory system process
Muscle contraction
- log10 (p-value)
F
Gene Ontology (BP) of MTJ depleted genes after exercise
Regulation of synpase structure or activity
Regulation of synpase organization
Positive regulation of synaptic transmission
Vesicle-mediated transport in synapse
Regulation of synaptic plasticity
Regulation of postsynaptic membrane potential
Postsynaptic organization
Synaptic vesiscle cycle
Neurotransmitter transport
Synapse assembly
- log10 (p-value)

Figure S7. Analyses related to MTJ translational profiling in exercised muscles

(A) Comparison of genes enriched in whole-muscle ribosomes (CK8-Cre–labeled) or MTJ ribosomes (Tigd4-Cre labeled) following exercise relative to sedentary conditions.

(B) Venn diagram showing overlap between translationally upregulated genes upon exercise in whole muscle and MTJ. The p-value was calculated using a hypergeometric test with a total background of 21,900 protein-coding genes in the mouse genome.

(C) Reactome pathway analysis of the commonly enriched genes identified in (D).

(D) Venn diagram showing overlap between translationally upregulated genes at the MTJ under sedentary and exercised conditions. The p-value was calculated using a hypergeometric test with a total background of 21,900 protein-coding genes in the mouse genome.

(E, F) Gene Ontology (biological process) analyses of genes enriched (E) or depleted (F) in MTJ-associated ribosomes following exercise.

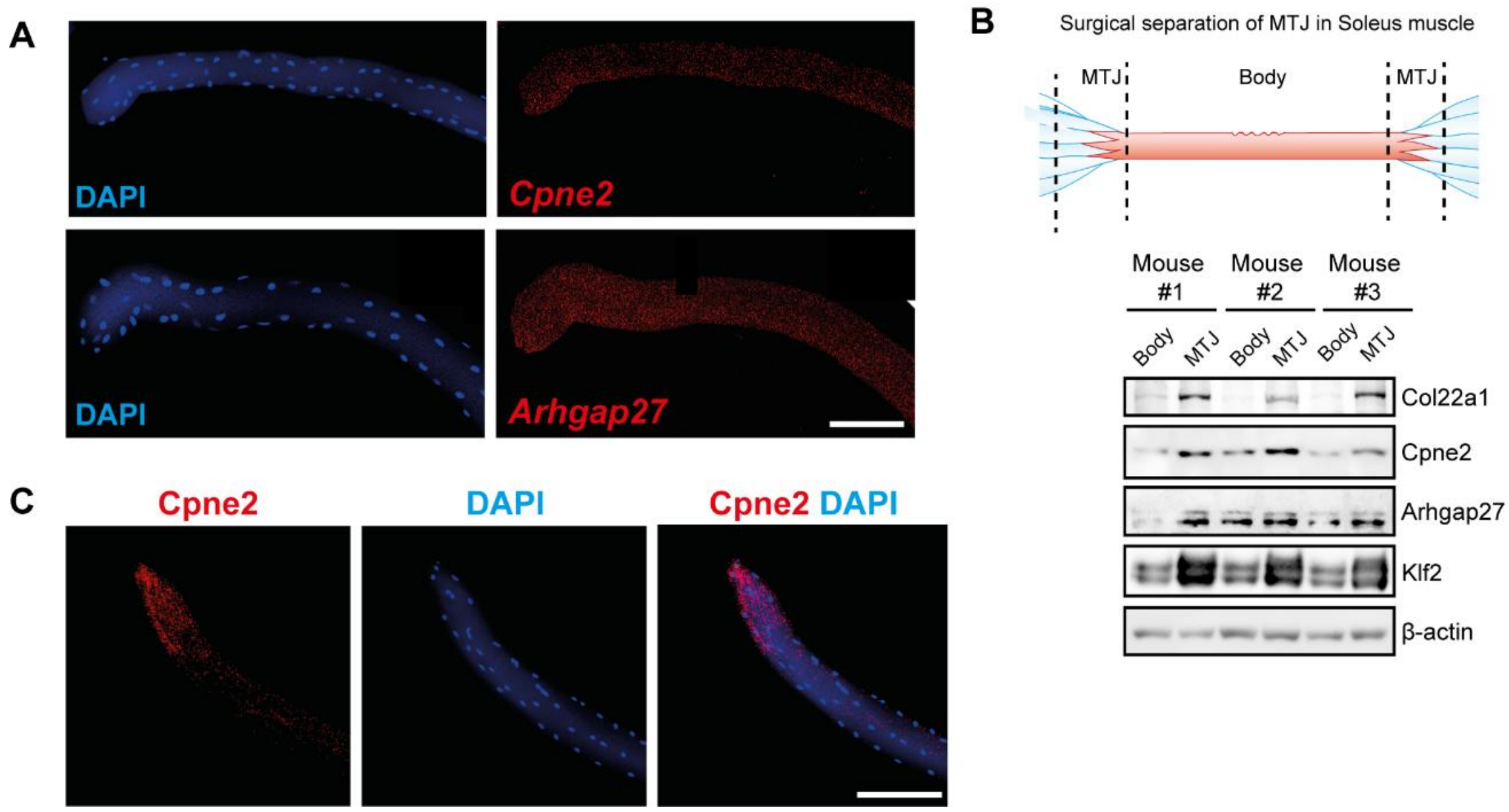

Figure S8. Validation of translational targets

(A) RNAscope of indicated transcripts in isolated EDL myofibers. Scale bar, 100 µm

(B) MTJ and non-MTJ parts of Soleus muscle were surgically separated and examined by Western blotting (n=3).

(C) Immunostaining of Cpne2 proteins in isolated EDL myofibers. Scale bar, 100 µm

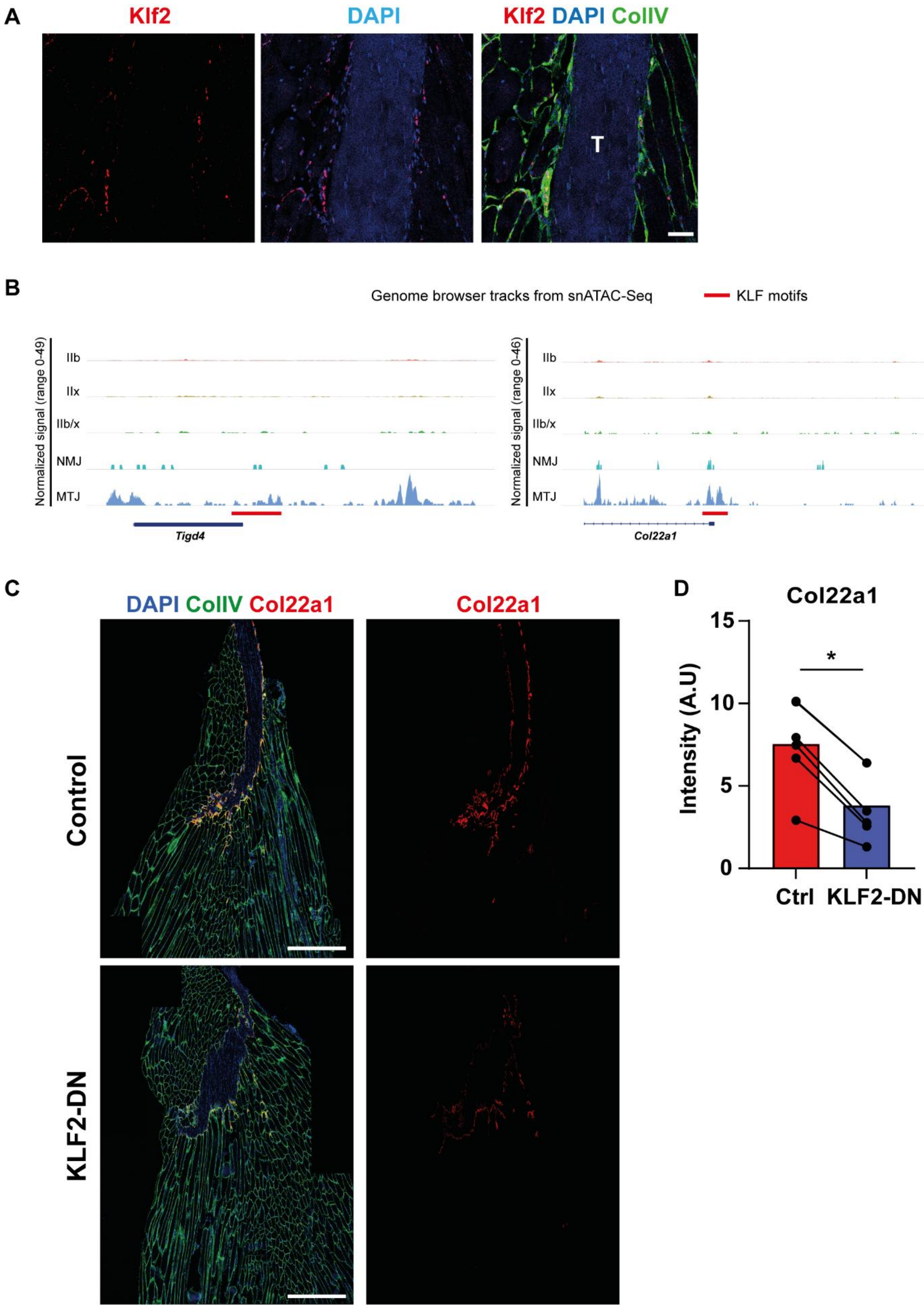
A
Klf2
DAPI
Klf2 DAPI CollV
T
B
Genome browser tracks from snATAC-Seq
KLF motifs
Normalized signal (range 0-49)
IIb
IIx
IIb/x
NMJ
MTJ
Tigd4
Normalized signal (range 0-46)
Col22a1
C
DAPI CollV Col22a1
Col22a1
Control
KLF2-DN
D
Col22a1
Intensity (A.U)
15
10
5
0
*
Ctrl
KLF2-DN

Figure S9. KLF factors control the expression of MTJ genes

(A) Longitudinal sections of TA muscle were immunostained with an anti-Klf2 antibody. Note the stronger expression of KLF2 along the MTJ. T, tendon. Scale bar, 100 µm

(B) Genome browser tracks of ATAC-Seq peaks at the *Tigd4* and *Col22a1* genes and location of KLF motifs.

(C) Reduced Col22a1 protein expression at the MTJ following KLF2-DN expression. Whole-muscle tile-scan images of TA sections stained for Col22a1 and Collagen IV are shown. All samples were acquired and processed under identical imaging conditions. Note that Col22a1 is specifically enriched at the MTJ, whereas Collagen IV is expressed throughout the muscle. Scale bars, 1000 µm.

(D) Quantification of (C) (n=5). Paired student's *t*-test, *, $P < 0.05$.

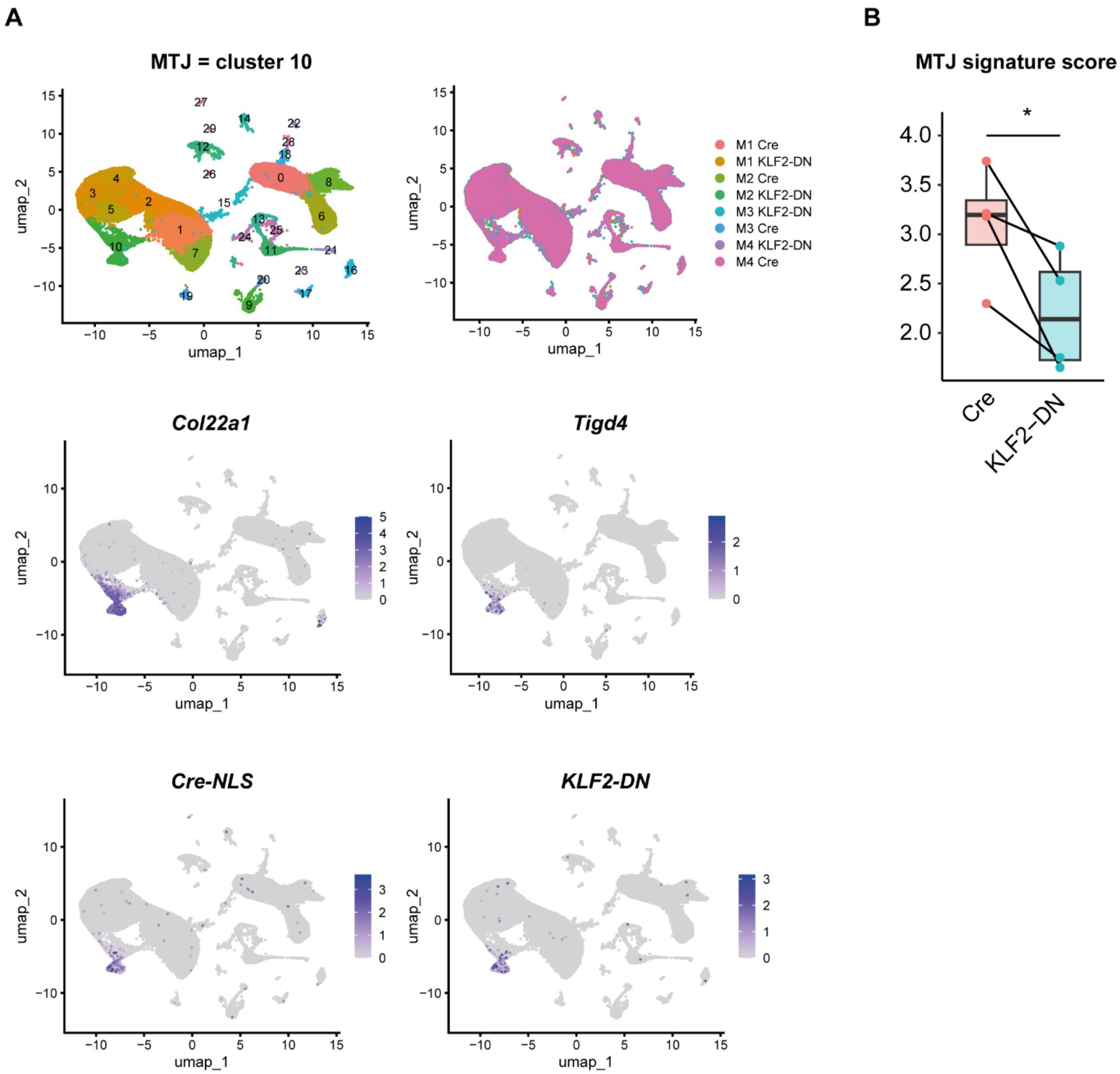

Figure S10. Global repression of MTJ gene signature by KLF2-DN revealed by snRNA-Seq of TA muscles.

(A) Integrated UMAP of TA muscles from four Cre-NLS injected (control) and four KLF2-DN injected mice. Cluster 10 corresponds to MTJ myonuclei, as indicated by the expression of *Tigd4* and *Col22a1*. *Cre-NLS* and *KLF2-DN* transcripts were also detected predominantly within the MTJ cluster.

(B) *Cre-NLS* positive and *KLF2-DN* positive nuclei were extracted, and the MTJ gene signature score was quantified. Paired student's *t*-test, *, $P < 0.05$.

Table S1. Genes up- and down-regulated in MTJ by bulk RNA-Seq in Figure 1G

Table S2. Full list of genes in the heatmap of Figure S6B

Table S3. Marker genes from RiboTag-Seq experiments

Sheet #1: Translationally induced genes in whole muscle (CK8) after exercise

Sheet #2: Translationally induced genes in MTJ after exercise

Sheet #3: Translationally enriched genes at the MTJ compared to whole muscle in sedentary condition

Sheet #4: Translationally enriched genes at the MTJ compared to whole muscle after exercise

Supplementary Video 1. Rotating video of 3D rendered image of myofiber in Figure 1C

Supplementary file 1. DNA sequence of AAV capsid used in this study
